# *catena*-Poly[[(4-methyl­benzoato-κ*O*)manganese(II)]-μ-aqua-bis­(μ-4-methyl­benzoato-κ^2^ *O*:*O*′)[(4-methyl­benzoato-κ*O*)manganese(II)]-bis­(μ-*N*,*N*-diethyl­nicotinamide)-κ^2^ *N* ^3^:*O*;*O*:*N* ^3^]

**DOI:** 10.1107/S1600536810020076

**Published:** 2010-06-05

**Authors:** Tuncer Hökelek, Hakan Dal, Barış Tercan, Efdal Çimen, Hacali Necefoğlu

**Affiliations:** a Department of Physics, Hacettepe University, 06800 Beytepe, Ankara, Turkey; b Department of Chemistry, Faculty of Science, Anadolu University, 26470 Yenibağlar, Eskişehir, Turkey; c Department of Physics, Karabük University, 78050, Karabük, Turkey; d Department of Chemistry, Kafkas University, 63100 Kars, Turkey

## Abstract

The asymmetric unit of the title complex, [Mn_2_(C_8_H_7_O_2_)_4_(C_10_H_14_N_2_O)_2_(H_2_O)]_*n*_, contains two crystallographically independent units. In each one, the Mn^II^ ions are bridged by two 4-methyl­benzoate (PMB) ligands and one water mol­ecule. In the crystal structure, each Mn^II^ ion is coordinated by three PMB ligands, one water mol­ecule and two symmetry-related *N*,*N*-diethyl­nicotinamide (DENA) ligands. Symmetry-related Mn^II^ ions are bridged by the N and O atoms of symmetry-related DENA ligands, forming polymeric chains parallel to [100]. The coord­ination environmnts for the Mn^II^ ions are slightly distorted octa­hedral. Intra­molecular O—H⋯O hydrogen bonds link bridging water mol­ecules to the carboxyl­ate O atoms of a neigh­boring polymeric chain. In the crystal structure, π–π contacts between benzene rings [centroid–centroid distance = 3.562 (1) Å] and weak C—H⋯π inter­actions are also observed.

## Related literature

For applications of transition-metal complexes with bio­chem­ically relevant ligands in biological systems, see: Antolini *et al.* (1982 ▶); Krishnamachari (1974 ▶). For the use of 4-amino­benzoic acids in coordination chemistry, see: Amiraslanov *et al.* (1979 ▶); Chen & Chen (2002 ▶); Hauptmann *et al.* (2000 ▶). *N*,*N*-Diethyl­nicotinamide (DENA) is an important respiratory stimulant, see: Bigoli *et al.* (1972 ▶). For structure–function–coordination relationships of the aryl­carboxyl­ate ion in Mn^II^ complexes of benzoic acid derivatives, see: Adiwidjaja *et al.* (1978 ▶); Antsyshkina *et al.* (1980 ▶); Catterick *et al.* (1974 ▶); Shnu­lin *et al.* (1981 ▶). For related structures, see: Hökelek *et al.* (2009*a* ▶,*b* ▶).
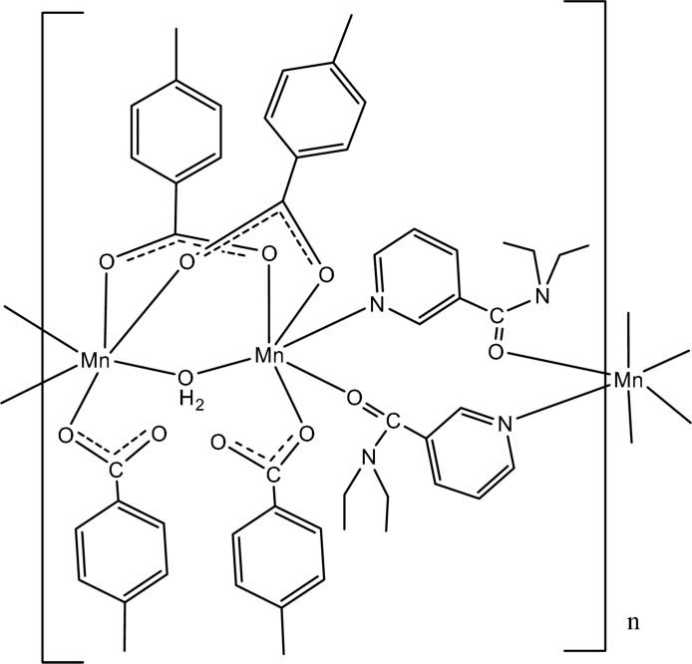


## Experimental

### 

#### Crystal data

[Mn_2_(C_8_H_7_O_2_)_4_(C_10_H_14_N_2_O)_2_(H_2_O)]
*M* *_r_* = 1024.90
Triclinic, 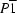

*a* = 10.5228 (2) Å
*b* = 19.1361 (3) Å
*c* = 26.6008 (4) Å
α = 70.537 (2)°
β = 78.836 (3)°
γ = 88.485 (3)°
*V* = 4950.63 (17) Å^3^
*Z* = 4
Mo *K*α radiation
μ = 0.57 mm^−1^
*T* = 100 K
0.35 × 0.24 × 0.15 mm

#### Data collection

Bruker Kappa APEXII CCD area-detector diffractometer
Absorption correction: multi-scan (*SADABS*; Bruker, 2005 ▶) *T*_min_ = 0.848, *T*_max_ = 0.917
87669 measured reflections
24563 independent reflections
17121 reflections with *I* > 2σ(*I*)
*R*_int_ = 0.049

#### Refinement

*R*[*F* ^2^ > 2σ(*F* ^2^)] = 0.042
*wR*(*F* ^2^) = 0.102
*S* = 1.02
24563 reflections
1275 parameters
1 restraint
H atoms treated by a mixture of independent and constrained refinement
Δρ_max_ = 0.40 e Å^−3^
Δρ_min_ = −0.51 e Å^−3^

### 

Data collection: *APEX2* (Bruker, 2007 ▶); cell refinement: *SAINT* (Bruker, 2007 ▶); data reduction: *SAINT*; program(s) used to solve structure: *SHELXS97* (Sheldrick, 2008 ▶); program(s) used to refine structure: *SHELXL97* (Sheldrick, 2008 ▶); molecular graphics: *Mercury* (Macrae *et al.*, 2006 ▶); software used to prepare material for publication: *WinGX* (Farrugia, 1999 ▶) and *PLATON* (Spek, 2009 ▶).

## Supplementary Material

Crystal structure: contains datablocks I, global. DOI: 10.1107/S1600536810020076/im2201sup1.cif

Structure factors: contains datablocks I. DOI: 10.1107/S1600536810020076/im2201Isup2.hkl

Additional supplementary materials:  crystallographic information; 3D view; checkCIF report

## Figures and Tables

**Table 1:** Hydrogen-bond geometry (Å, °) *Cg*1, *Cg*5, *Cg*10 and *Cg*12 are the centroids of the C2–C7, N1/C33–C37, C78–C83 and N6/C90–C94 rings, respectively.

*D*—H⋯*A*	*D*—H	H⋯*A*	*D*⋯*A*	*D*—H⋯*A*
O9—H9*A*⋯O7	0.94 (3)	1.62 (3)	2.552 (2)	170 (3)
O9—H9*B*⋯O5	0.94 (2)	1.59 (2)	2.520 (2)	173 (3)
O22—H22*A*⋯O16	0.92 (3)	1.64 (3)	2.544 (2)	166 (2)
O22—H22*B*⋯O18	0.97 (4)	1.60 (4)	2.558 (2)	169 (3)
C52—H52*A*⋯*Cg*12^i^	0.96	2.87	3.782 (3)	160
C60—H60*C*⋯*Cg*1^ii^	0.96	2.99	3.841 (3)	149
C84—H84*B*⋯*Cg*10^iii^	0.96	2.88	3.609 (3)	134
C104—H10*E*⋯*Cg*5^iv^	0.96	2.82	3.709 (3)	155

Symmetry codes: (i) 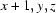; (ii) 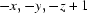; (iii) 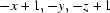; (iv) 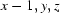.

**Fig. 1.**
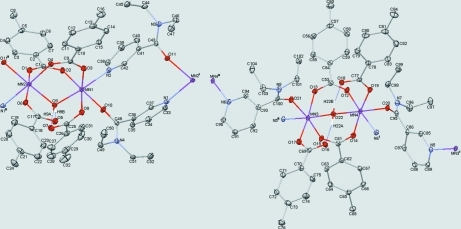
The molecular structure of the title molecule with the atom-numbering scheme. Displacement ellipsoids are drawn at the 35% probability level. Hydrogen bonds are shown as dashed lines. Hydrogen atoms not involved in hydrogen bonding are omitted for clarity. Primed atoms are generated by the symmetry operators:(') 1 + x, y, z, ('') x - 1, y, z.

**Fig. 2.**
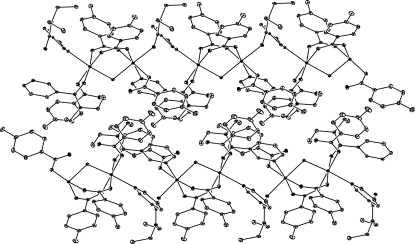
Part of the polymeric chains of the title compound.

## supplementary crystallographic information

### Comment

Transition metal complexes with biochemicaly relevant ligands show interesting physical and/or chemical properties, through which they may find applications in biological systems (Antolini *et al.*, 1982). Some benzoic acid derivatives, such as 4-aminobenzoic acid, have been extensively reported in coordination chemistry as bifunctional organic ligands due to the varieties of their coordination modes (Chen & Chen, 2002; Amiraslanov *et al.*, 1979; Hauptmann *et al.*, 2000). Nicotinamide (NA) is one form of niacin. A deficiency of this vitamin leads to loss of copper from the body, known as pellagra disease. Victims of pellagra show unusually high serum and urinary copper levels (Krishnamachari, 1974). The nicotinic acid derivative *N*,*N*-Diethylnicotinamide (DENA) is an important respiratory stimulant (Bigoli *et al.*, 1972).

The structure-function-coordination relationships of arylcarboxylate ions in Mn^II^ complexes of benzoic acid derivatives may also change depending on the nature and position of the substituted groups on the benzene ring, the nature of the additional ligand molecule or solvent, and the pH and temperature of synthesis (Shnulin *et al.*, 1981; Antsyshkina *et al.*, 1980; Adiwidjaja *et al.*, 1978). When pyridine and its derivatives are used instead of water molecules, the structure is completely different (Catterick *et al.*, 1974). The title complex, (I), was synthesized and its crystal structure is reported herein.

The asymmetric unit of the title complex, (I), contains two crystallographically independent moieties. In each moiety, Mn^II^ ions are bridged by two 4-methylbenzoate (PMB) ligands and one water molecule. In the crystal structure of the title complex, each Mn^II^ ion is coordinated by three PMB ligands, one water molecule and two symmetry related DENA ligands. Symmetry related Mn^II^ ions are bridged by the N and O atoms of symmetry related DENA ligands forming polymeric chains (Fig. 1).

Coordination spheres around Mn1 and Mn2 (corresponding to Mn3 and Mn4 in the second moiety) are different. In the octahedron around Mn1 the nitrogen atom of a DENA ligand is in *trans*-position with respect to the bridging water ligand. The monodentate 4-methylbenzoate oxygen and another oxygen from the DENA ligand are *trans* to the bridging 4-methylbenzoate ligands. Around Mn2 bridging 4-methylbenzoate ligands are *trans* with respect to a DENA nitrogen atom and an oxygen from a another monodentate 4-methylbenzoate ligand.

Atoms (O2, O3, O8, O10), (O1, O4, O6, N1''), (O13, O15, O17, O21) and (O12, O14, O19, N6') in the equatorial planes around Mn1, Mn2, Mn3 and Mn4, respectively, form slightly distorted square-planar arrangements, while the slightly distorted octahedral coordinations are completed by the atoms (O9, N2), (O9, O11''), (O22, N5'') and (O20, O22) in the axial positions (Table 1 and Fig. 1) [symmetry codes:(') 1 + x, y, z, ('') x - 1, y, z]. The structures of similar Mn^II^ and Zn(II) polymeric complexes {[Mn(C_11_H_14_NO_2_)_2_(H_2_O)_3_].2H_2_O}_n_ (Hökelek *et al.*, 2009a) and [Zn(C_8_H_5_O_3_)_2_(C_6_H_6_N_2_O)]_n_ (Hökelek *et al.*, 2009b) have also been determined.

The average Mn—O bond length (Table 1) is 2.1699 (14) Å and Mn atoms are displaced out of the least-squares planes of the carboxylate groups: Mn1 atom for (O1/C1/O2), (O3/C9/O4) and (O7/C25/O8) by 0.3349 (3), 0.2642 (4) and -0.5844 (4) Å, respectively, Mn2 atom for (O1/C1/O2), (O3/C9/O4) and (O5/C17/O6) by -0.0412 (3), 0.4940 (4) and 0.1143 (3) Å, respectively, Mn3 atom for (O12/C53/O13), (O14/C61/O15) and (O16/C69/O17) by 0.3463 (4), -0.2062 (3) and 0.2611 (4) Å, respectively, and Mn4 atom for (O12/C53/O13), (O14/C61/O15) and (O18/C77/O19) by 0.6423 (4), 0.0305 (3) and 0.3320 (3) Å, respectively. The dihedral angles between the planar carboxylate groups and the adjacent benzene rings A (C2—C7), B (C10—C15), C (C18—C23), D (C26—C31), G (C54—C59), H (C62—C67), I (C70—C75) and J (C78—C83) are 13.35 (16), 10.82 (12), 7.48 (15), 7.33 (9), 11.86 (10), 11.42 (10), 6.68 (18) and 1.79 (11) °, respectively, while those between rings A, B, C, D, E (N1/C33—C37), F (N2/C38—C42) and G, H, I, J, K (N5/C86—C89), L (N6/C90—C94) are A/B = 64.86 (7), A/C = 35.09 (7), A/D = 63.93 (9), B/C = 83.44 (7), B/D = 22.10 (8), C/D = 87.56 (8), E/F = 33.93 (6) ° and G/H = 60.41 (6), G/I = 14.54 (8), G/J = 62.80 (7), H/I = 68.14 (7), H/J = 23.40 (7), I/J = 75.02 (8), K/L = 43.80 (8) °.

Intramolecular O—H···O hydrogen bonds (Table 1) link bridging water molecules to carboxylate O atoms of neighboring polymeric chains. In the crystal structure, π–π contacts between the benzene rings, Cg3—Cg3^i^ with a centroid-centroid distance of 3.562 (1) Å [symmetry code: (i) -x, 1 - y, -z, where Cg3 is the centroid of the ring C (C18—C23)] and four weak C—H···π interactions are also observed (Table 1).

### Experimental

The title compound was prepared by the reaction of MnSO_4_.H_2_O (0.84 g, 5 mmol) in H_2_O (10 ml) and DENA (1.78 g, 10 mmol) in H_2_O (10 ml) with sodium 4-methylbenzoate (1.58 g, 10 mmol) in H_2_O (150 ml). The mixture was filtered and set aside to crystallize at ambient temperature for one week, giving colorless single crystals (yield 2.05 g, 40.02 %).

### Refinement

Atoms H9A, H9B, H22A and H22B (for water molecules) were located in difference Fourier maps and refined isotropically, with restrain of O9—H9B = 0.937 (18) Å. [U_iso_(H) = 0.046 (8), 0.069 (11), 0.035 (7) and 0.081 (12) Å^2^ for H9A, H9B, H22A and H22B, respectively]. The remaining H atoms were positioned geometrically with C—H = 0.93, 0.97 and 0.96 Å for aromatic, methylene and methyl H atoms, respectively, and constrained to ride on their parent atoms, with U_iso_(H) = xU_eq_(C), where x = 1.5 for methyl H and x = 1.2 for all other H atoms.

### Crystal data

**Table:**

[Mn_2_(C_8_H_7_O_2_)_4_(C_10_H_14_N_2_O)_2_(H_2_O)]	*Z* = 4
*M**_r_* = 1024.90	*F*(000) = 2144
Triclinic, *P*1	*D*_x_ = 1.375 Mg m^−^^3^
Hall symbol: -P 1	Mo *K*α radiation, λ = 0.71073 Å
*a* = 10.5228 (2) Å	Cell parameters from 9867 reflections
*b* = 19.1361 (3) Å	θ = 2.3–28.0°
*c* = 26.6008 (4) Å	µ = 0.57 mm^−^^1^
α = 70.537 (2)°	*T* = 100 K
β = 78.836 (3)°	Block, colorless
γ = 88.485 (3)°	0.35 × 0.24 × 0.15 mm
*V* = 4950.63 (17) Å^3^	

### Data collection

**Table:**

Bruker Kappa APEXII CCD area-detector diffractometer	24563 independent reflections
Radiation source: fine-focus sealed tube	17121 reflections with *I* > 2σ(*I*)
graphite	*R*_int_ = 0.049
φ and ω scans	θ_max_ = 28.4°, θ_min_ = 1.7°
Absorption correction: multi-scan (*SADABS*; Bruker, 2005)	*h* = −14→14
*T*_min_ = 0.848, *T*_max_ = 0.917	*k* = −25→25
87669 measured reflections	*l* = −35→35

### Refinement

**Table:**

Refinement on *F*^2^	Primary atom site location: structure-invariant direct methods
Least-squares matrix: full	Secondary atom site location: difference Fourier map
*R*[*F*^2^ > 2σ(*F*^2^)] = 0.042	Hydrogen site location: inferred from neighbouring sites
*wR*(*F*^2^) = 0.102	H atoms treated by a mixture of independent and constrained refinement
*S* = 1.02	*w* = 1/[σ^2^(*F*_o_^2^) + (0.0376*P*)^2^ + 2.5004*P*] where *P* = (*F*_o_^2^ + 2*F*_c_^2^)/3
24563 reflections	(Δ/σ)_max_ = 0.001
1275 parameters	Δρ_max_ = 0.40 e Å^−^^3^
1 restraint	Δρ_min_ = −0.51 e Å^−^^3^

### Special details

**Table:**

Geometry. All esds (except the esd in the dihedral angle between two l.s. planes) are estimated using the full covariance matrix. The cell esds are taken into account individually in the estimation of esds in distances, angles and torsion angles; correlations between esds in cell parameters are only used when they are defined by crystal symmetry. An approximate (isotropic) treatment of cell esds is used for estimating esds involving l.s. planes.
Refinement. Refinement of *F*^2^ against ALL reflections. The weighted *R*-factor wR and goodness of fit *S* are based on *F*^2^, conventional *R*-factors *R* are based on *F*, with *F* set to zero for negative *F*^2^. The threshold expression of *F*^2^ > σ(*F*^2^) is used only for calculating *R*-factors(gt) etc. and is not relevant to the choice of reflections for refinement. *R*-factors based on *F*^2^ are statistically about twice as large as those based on *F*, and *R*- factors based on ALL data will be even larger.

### Fractional atomic coordinates and isotropic or equivalent isotropic displacement parameters (Å^2^)

**Table:**

	*x*	*y*	*z*	*U*_iso_*/*U*_eq_	
Mn1	0.29102 (3)	0.092307 (17)	0.156751 (12)	0.01457 (7)	
Mn2	−0.02201 (3)	0.133661 (17)	0.112030 (12)	0.01425 (7)	
Mn3	0.20220 (3)	0.461244 (17)	0.326281 (12)	0.01420 (7)	
Mn4	0.51449 (3)	0.407633 (17)	0.366892 (12)	0.01429 (7)	
O1	−0.00798 (14)	0.01586 (8)	0.14766 (6)	0.0216 (3)	
O2	0.19077 (14)	−0.00928 (8)	0.16623 (6)	0.0215 (3)	
O3	0.32961 (14)	0.13578 (8)	0.06967 (6)	0.0203 (3)	
O4	0.13544 (14)	0.15161 (8)	0.04538 (6)	0.0210 (3)	
O5	0.10170 (14)	0.28937 (8)	0.11920 (6)	0.0223 (3)	
O6	−0.05798 (14)	0.25125 (8)	0.08837 (6)	0.0191 (3)	
O7	0.05950 (15)	0.08986 (9)	0.26520 (6)	0.0245 (3)	
O8	0.26278 (14)	0.05493 (9)	0.24495 (6)	0.0220 (3)	
O9	0.11046 (14)	0.15084 (8)	0.16200 (6)	0.0164 (3)	
H9A	0.085 (3)	0.1322 (15)	0.1999 (12)	0.046 (8)*	
H9B	0.112 (3)	0.2027 (10)	0.1476 (13)	0.069 (11)*	
O10	0.40261 (14)	0.18976 (8)	0.15298 (6)	0.0210 (3)	
O11	−0.15129 (14)	0.11306 (8)	0.06165 (6)	0.0183 (3)	
O12	0.35950 (14)	0.37541 (9)	0.43431 (6)	0.0217 (3)	
O13	0.16363 (14)	0.39528 (8)	0.41208 (6)	0.0193 (3)	
O14	0.49611 (14)	0.52498 (8)	0.34726 (6)	0.0222 (3)	
O15	0.30051 (14)	0.55792 (8)	0.32796 (6)	0.0205 (3)	
O16	0.42449 (14)	0.47310 (9)	0.21309 (6)	0.0236 (3)	
O17	0.22779 (14)	0.51251 (8)	0.23789 (6)	0.0217 (3)	
O18	0.38854 (15)	0.25966 (8)	0.35204 (6)	0.0243 (4)	
O19	0.55809 (14)	0.29317 (8)	0.37939 (6)	0.0207 (3)	
O20	0.65101 (13)	0.41069 (8)	0.41920 (6)	0.0187 (3)	
O21	0.08786 (14)	0.37412 (8)	0.31793 (6)	0.0217 (3)	
O22	0.37934 (14)	0.40100 (9)	0.31456 (6)	0.0160 (3)	
H22A	0.406 (2)	0.4217 (14)	0.2775 (11)	0.035 (7)*	
H22B	0.374 (3)	0.347 (2)	0.3269 (14)	0.081 (12)*	
N1	−0.20574 (17)	0.12154 (10)	0.17872 (7)	0.0171 (4)	
N2	0.47393 (17)	0.02732 (10)	0.14640 (7)	0.0172 (4)	
N3	0.83418 (17)	0.05090 (10)	0.00421 (7)	0.0181 (4)	
N4	0.45429 (17)	0.27187 (10)	0.19043 (7)	0.0195 (4)	
N5	0.01818 (16)	0.51791 (10)	0.35032 (7)	0.0166 (4)	
N6	0.68991 (16)	0.43439 (9)	0.29721 (7)	0.0168 (4)	
N7	0.65443 (17)	0.44424 (10)	0.49232 (7)	0.0193 (4)	
N8	0.03462 (17)	0.29462 (10)	0.27868 (7)	0.0179 (4)	
C1	0.0784 (2)	−0.02758 (12)	0.16489 (8)	0.0169 (4)	
C2	0.0427 (2)	−0.10932 (11)	0.18415 (8)	0.0169 (4)	
C3	−0.0853 (2)	−0.13269 (12)	0.19115 (8)	0.0186 (4)	
H3	−0.1495	−0.0980	0.1870	0.022*	
C4	−0.1182 (2)	−0.20697 (12)	0.20415 (9)	0.0208 (5)	
H4	−0.2045	−0.2217	0.2088	0.025*	
C5	−0.0241 (2)	−0.25999 (12)	0.21033 (9)	0.0208 (5)	
C6	0.1028 (2)	−0.23687 (12)	0.20604 (9)	0.0216 (5)	
H6	0.1662	−0.2718	0.2120	0.026*	
C7	0.1363 (2)	−0.16242 (12)	0.19306 (9)	0.0201 (5)	
H7	0.2218	−0.1479	0.1903	0.024*	
C8	−0.0587 (2)	−0.33925 (12)	0.21777 (10)	0.0281 (5)	
H8A	0.0082	−0.3707	0.2316	0.042*	
H8B	−0.0667	−0.3431	0.1834	0.042*	
H8C	−0.1395	−0.3545	0.2431	0.042*	
C9	0.2572 (2)	0.15832 (11)	0.03538 (8)	0.0165 (4)	
C10	0.3218 (2)	0.19464 (11)	−0.02273 (8)	0.0171 (4)	
C11	0.2489 (2)	0.23152 (12)	−0.06148 (9)	0.0210 (5)	
H11	0.1593	0.2321	−0.0513	0.025*	
C12	0.3087 (2)	0.26728 (13)	−0.11504 (9)	0.0248 (5)	
H12	0.2587	0.2920	−0.1403	0.030*	
C13	0.4422 (2)	0.26688 (12)	−0.13162 (9)	0.0233 (5)	
C14	0.5145 (2)	0.22896 (13)	−0.09326 (9)	0.0238 (5)	
H14	0.6037	0.2270	−0.1037	0.029*	
C15	0.4551 (2)	0.19386 (12)	−0.03941 (9)	0.0201 (5)	
H15	0.5054	0.1695	−0.0142	0.024*	
C16	0.5066 (3)	0.30636 (14)	−0.18978 (9)	0.0347 (6)	
H16A	0.4432	0.3328	−0.2097	0.052*	
H16B	0.5447	0.2708	−0.2059	0.052*	
H16C	0.5728	0.3407	−0.1909	0.052*	
C17	0.0039 (2)	0.30073 (11)	0.09722 (8)	0.0171 (4)	
C18	−0.0438 (2)	0.37802 (12)	0.08131 (8)	0.0178 (4)	
C19	−0.1414 (2)	0.39756 (13)	0.05159 (10)	0.0274 (5)	
H19	−0.1782	0.3622	0.0412	0.033*	
C20	−0.1848 (2)	0.46928 (13)	0.03717 (10)	0.0298 (5)	
H20	−0.2504	0.4813	0.0172	0.036*	
C21	−0.1322 (2)	0.52330 (13)	0.05200 (9)	0.0260 (5)	
C22	−0.0330 (2)	0.50357 (13)	0.08122 (10)	0.0281 (5)	
H22	0.0050	0.5391	0.0911	0.034*	
C23	0.0104 (2)	0.43205 (12)	0.09590 (9)	0.0231 (5)	
H23	0.0764	0.4200	0.1157	0.028*	
C24	−0.1783 (2)	0.60127 (13)	0.03638 (11)	0.0342 (6)	
H24A	−0.1075	0.6352	0.0311	0.051*	
H24B	−0.2463	0.6057	0.0648	0.051*	
H24C	−0.2105	0.6127	0.0033	0.051*	
C25	0.1568 (2)	0.05343 (12)	0.27750 (9)	0.0187 (4)	
C26	0.1452 (2)	0.00386 (12)	0.33574 (9)	0.0204 (5)	
C27	0.0350 (2)	0.00399 (14)	0.37437 (9)	0.0300 (5)	
H27	−0.0304	0.0362	0.3645	0.036*	
C28	0.0225 (3)	−0.04345 (15)	0.42726 (10)	0.0391 (7)	
H28	−0.0509	−0.0419	0.4527	0.047*	
C29	0.1168 (3)	−0.09347 (14)	0.44339 (10)	0.0361 (7)	
C30	0.2254 (3)	−0.09338 (14)	0.40479 (11)	0.0367 (7)	
H30	0.2900	−0.1264	0.4146	0.044*	
C31	0.2404 (2)	−0.04492 (13)	0.35141 (10)	0.0281 (5)	
H31	0.3150	−0.0455	0.3263	0.034*	
C32	0.0996 (3)	−0.14617 (17)	0.50102 (11)	0.0553 (9)	
H32A	0.0816	−0.1187	0.5257	0.083*	
H32B	0.1775	−0.1726	0.5060	0.083*	
H32C	0.0286	−0.1808	0.5080	0.083*	
C33	−0.2241 (2)	0.07042 (11)	0.22842 (9)	0.0183 (4)	
H33	−0.1566	0.0398	0.2383	0.022*	
C34	−0.3392 (2)	0.06135 (12)	0.26569 (9)	0.0214 (5)	
H34	−0.3481	0.0256	0.3000	0.026*	
C35	−0.4409 (2)	0.10575 (12)	0.25143 (9)	0.0199 (5)	
H35	−0.5198	0.0997	0.2756	0.024*	
C36	−0.42287 (19)	0.15955 (11)	0.20030 (8)	0.0155 (4)	
C37	−0.30381 (19)	0.16563 (11)	0.16559 (8)	0.0161 (4)	
H37	−0.2914	0.2021	0.1315	0.019*	
C38	0.4733 (2)	−0.04641 (12)	0.17108 (9)	0.0190 (4)	
H38	0.4031	−0.0693	0.1985	0.023*	
C39	0.5722 (2)	−0.08989 (12)	0.15759 (9)	0.0213 (5)	
H39	0.5682	−0.1408	0.1756	0.026*	
C40	0.6768 (2)	−0.05651 (12)	0.11698 (9)	0.0211 (5)	
H40	0.7433	−0.0848	0.1066	0.025*	
C41	0.6813 (2)	0.02013 (11)	0.09173 (8)	0.0164 (4)	
C42	0.5773 (2)	0.05918 (11)	0.10813 (8)	0.0160 (4)	
H42	0.5799	0.1104	0.0915	0.019*	
C43	0.79495 (19)	0.06378 (11)	0.05116 (8)	0.0159 (4)	
C44	0.7643 (2)	0.00135 (13)	−0.01427 (9)	0.0240 (5)	
H44A	0.7213	−0.0388	0.0173	0.029*	
H44B	0.8267	−0.0203	−0.0362	0.029*	
C45	0.6646 (2)	0.03952 (14)	−0.04705 (10)	0.0317 (6)	
H45A	0.6249	0.0046	−0.0587	0.047*	
H45B	0.7061	0.0796	−0.0783	0.047*	
H45C	0.5995	0.0586	−0.0250	0.047*	
C46	0.9449 (2)	0.09632 (12)	−0.03440 (9)	0.0228 (5)	
H46A	0.9768	0.0736	−0.0618	0.027*	
H46B	1.0140	0.0968	−0.0151	0.027*	
C47	0.9114 (2)	0.17535 (13)	−0.06227 (10)	0.0287 (5)	
H47A	0.9843	0.2008	−0.0896	0.043*	
H47B	0.8905	0.2002	−0.0359	0.043*	
H47C	0.8383	0.1753	−0.0789	0.043*	
C48	0.47036 (19)	0.20863 (12)	0.18015 (8)	0.0162 (4)	
C49	0.3529 (2)	0.32107 (13)	0.16928 (10)	0.0244 (5)	
H49A	0.3339	0.3552	0.1895	0.029*	
H49B	0.2745	0.2913	0.1756	0.029*	
C50	0.3893 (2)	0.36527 (14)	0.10934 (10)	0.0342 (6)	
H50A	0.3243	0.4005	0.0993	0.051*	
H50B	0.3954	0.3323	0.0886	0.051*	
H50C	0.4714	0.3911	0.1020	0.051*	
C51	0.5357 (2)	0.29619 (13)	0.22146 (9)	0.0248 (5)	
H51A	0.5711	0.2529	0.2448	0.030*	
H51B	0.4816	0.3197	0.2446	0.030*	
C52	0.6457 (2)	0.34963 (14)	0.18583 (11)	0.0342 (6)	
H52A	0.6961	0.3623	0.2082	0.051*	
H52B	0.6113	0.3937	0.1639	0.051*	
H52C	0.6997	0.3268	0.1627	0.051*	
C53	0.2385 (2)	0.36444 (11)	0.44399 (8)	0.0167 (4)	
C54	0.1792 (2)	0.31129 (11)	0.49916 (8)	0.0170 (4)	
C55	0.0468 (2)	0.30495 (12)	0.51870 (9)	0.0204 (5)	
H55	−0.0082	0.3327	0.4968	0.024*	
C56	−0.0045 (2)	0.25787 (13)	0.57045 (9)	0.0240 (5)	
H56	−0.0938	0.2550	0.5829	0.029*	
C57	0.0741 (2)	0.21472 (12)	0.60428 (9)	0.0238 (5)	
C58	0.2070 (2)	0.21989 (12)	0.58382 (9)	0.0232 (5)	
H58	0.2618	0.1908	0.6052	0.028*	
C59	0.2591 (2)	0.26750 (12)	0.53229 (9)	0.0219 (5)	
H59	0.3483	0.2703	0.5196	0.026*	
C60	0.0182 (3)	0.16500 (14)	0.66114 (10)	0.0340 (6)	
H60A	0.0653	0.1203	0.6696	0.051*	
H60B	−0.0713	0.1529	0.6637	0.051*	
H60C	0.0250	0.1901	0.6864	0.051*	
C61	0.4106 (2)	0.57161 (12)	0.33415 (8)	0.0165 (4)	
C62	0.4477 (2)	0.65083 (11)	0.32589 (8)	0.0158 (4)	
C63	0.3573 (2)	0.70589 (12)	0.32052 (9)	0.0202 (5)	
H63	0.2711	0.6941	0.3220	0.024*	
C64	0.3946 (2)	0.77828 (12)	0.31296 (9)	0.0220 (5)	
H64	0.3330	0.8145	0.3095	0.026*	
C65	0.5228 (2)	0.79759 (12)	0.31052 (9)	0.0200 (5)	
C66	0.6129 (2)	0.74237 (12)	0.31522 (9)	0.0202 (5)	
H66	0.6992	0.7541	0.3134	0.024*	
C67	0.5759 (2)	0.67020 (12)	0.32261 (8)	0.0183 (4)	
H67	0.6378	0.6342	0.3254	0.022*	
C68	0.5636 (2)	0.87545 (12)	0.30437 (10)	0.0278 (5)	
H68A	0.5172	0.9104	0.2800	0.042*	
H68B	0.6550	0.8835	0.2900	0.042*	
H68C	0.5446	0.8821	0.3392	0.042*	
C69	0.3274 (2)	0.51141 (12)	0.20306 (9)	0.0179 (4)	
C70	0.3331 (2)	0.56018 (12)	0.14482 (8)	0.0190 (4)	
C71	0.2283 (2)	0.60070 (12)	0.12891 (9)	0.0229 (5)	
H71	0.1515	0.5965	0.1540	0.027*	
C72	0.2373 (2)	0.64770 (13)	0.07557 (10)	0.0272 (5)	
H72	0.1654	0.6738	0.0654	0.033*	
C73	0.3505 (2)	0.65661 (13)	0.03725 (9)	0.0272 (5)	
C74	0.4538 (2)	0.61449 (14)	0.05329 (9)	0.0304 (6)	
H74	0.5302	0.6182	0.0280	0.036*	
C75	0.4457 (2)	0.56714 (13)	0.10592 (9)	0.0255 (5)	
H75	0.5166	0.5395	0.1155	0.031*	
C76	0.3644 (3)	0.71158 (14)	−0.01987 (10)	0.0355 (6)	
H76A	0.4412	0.7426	−0.0283	0.053*	
H76B	0.2900	0.7418	−0.0223	0.053*	
H76C	0.3709	0.6852	−0.0452	0.053*	
C77	0.4867 (2)	0.24556 (12)	0.37353 (8)	0.0185 (4)	
C78	0.5223 (2)	0.16569 (12)	0.39390 (9)	0.0198 (5)	
C79	0.4467 (2)	0.11043 (12)	0.38984 (9)	0.0245 (5)	
H79	0.3736	0.1228	0.3744	0.029*	
C80	0.4790 (2)	0.03679 (13)	0.40862 (10)	0.0287 (5)	
H80	0.4263	0.0004	0.4060	0.034*	
C81	0.5883 (2)	0.01624 (13)	0.43122 (10)	0.0277 (5)	
C82	0.6634 (3)	0.07218 (14)	0.43503 (11)	0.0346 (6)	
H82	0.7368	0.0600	0.4503	0.042*	
C83	0.6311 (2)	0.14594 (13)	0.41647 (10)	0.0289 (5)	
H83	0.6833	0.1824	0.4193	0.035*	
C84	0.6237 (3)	−0.06375 (13)	0.45167 (11)	0.0393 (7)	
H84A	0.5581	−0.0947	0.4481	0.059*	
H84B	0.6300	−0.0773	0.4892	0.059*	
H84C	0.7055	−0.0702	0.4307	0.059*	
C85	−0.08494 (19)	0.47597 (12)	0.38283 (8)	0.0156 (4)	
H85	−0.0835	0.4250	0.3899	0.019*	
C86	−0.19401 (19)	0.50452 (12)	0.40655 (8)	0.0159 (4)	
C87	−0.1961 (2)	0.58115 (12)	0.39485 (9)	0.0196 (5)	
H87	−0.2671	0.6026	0.4099	0.023*	
C88	−0.0908 (2)	0.62472 (12)	0.36043 (9)	0.0211 (5)	
H88	−0.0906	0.6760	0.3515	0.025*	
C89	0.0139 (2)	0.59133 (12)	0.33947 (9)	0.0186 (4)	
H89	0.0848	0.6211	0.3167	0.022*	
C90	0.7032 (2)	0.48943 (11)	0.24927 (8)	0.0180 (4)	
H90	0.6323	0.5181	0.2412	0.022*	
C91	0.8177 (2)	0.50526 (12)	0.21137 (9)	0.0211 (5)	
H91	0.8227	0.5434	0.1783	0.025*	
C92	0.9247 (2)	0.46414 (12)	0.22288 (9)	0.0195 (4)	
H92	1.0036	0.4752	0.1985	0.023*	
C93	0.9114 (2)	0.40577 (11)	0.27179 (8)	0.0159 (4)	
C94	0.79259 (19)	0.39322 (11)	0.30731 (8)	0.0160 (4)	
H94	0.7838	0.3540	0.3400	0.019*	
C95	0.69637 (19)	0.45079 (12)	0.44036 (8)	0.0160 (4)	
C96	0.7140 (2)	0.48486 (13)	0.52104 (9)	0.0246 (5)	
H96A	0.6495	0.4891	0.5512	0.030*	
H96B	0.7400	0.5346	0.4963	0.030*	
C97	0.8309 (2)	0.44791 (14)	0.54251 (10)	0.0290 (5)	
H97A	0.8634	0.4759	0.5617	0.044*	
H97B	0.8972	0.4460	0.5127	0.044*	
H97C	0.8062	0.3984	0.5668	0.044*	
C98	0.5546 (2)	0.38594 (13)	0.52469 (9)	0.0257 (5)	
H98A	0.4857	0.3888	0.5046	0.031*	
H98B	0.5175	0.3950	0.5582	0.031*	
C99	0.6063 (2)	0.30848 (14)	0.53846 (10)	0.0317 (6)	
H99A	0.5389	0.2732	0.5620	0.048*	
H99B	0.6779	0.3061	0.5564	0.048*	
H99C	0.6348	0.2971	0.5056	0.048*	
C100	0.01980 (19)	0.35729 (12)	0.29004 (8)	0.0156 (4)	
C101	0.1292 (2)	0.24234 (12)	0.30289 (9)	0.0228 (5)	
H10A	0.2107	0.2691	0.2970	0.027*	
H10B	0.1446	0.2056	0.2848	0.027*	
C102	0.0828 (2)	0.20352 (14)	0.36308 (10)	0.0324 (6)	
H10H	0.1444	0.1678	0.3768	0.049*	
H10I	0.0003	0.1787	0.3692	0.049*	
H10J	0.0742	0.2393	0.3815	0.049*	
C103	−0.0447 (2)	0.27293 (13)	0.24597 (9)	0.0232 (5)	
H10C	0.0103	0.2505	0.2223	0.028*	
H10D	−0.0790	0.3171	0.2231	0.028*	
C104	−0.1561 (2)	0.21903 (13)	0.27990 (10)	0.0298 (6)	
H10E	−0.2054	0.2081	0.2564	0.045*	
H10F	−0.2109	0.2407	0.3036	0.045*	
H10G	−0.1227	0.1741	0.3012	0.045*	

### Atomic displacement parameters (Å^2^)

**Table:**

	*U*^11^	*U*^22^	*U*^33^	*U*^12^	*U*^13^	*U*^23^
Mn1	0.01369 (16)	0.01606 (16)	0.01613 (16)	0.00008 (12)	−0.00361 (12)	−0.00779 (13)
Mn2	0.01361 (16)	0.01591 (16)	0.01577 (16)	0.00055 (12)	−0.00323 (12)	−0.00843 (13)
Mn3	0.01195 (15)	0.01724 (16)	0.01632 (16)	0.00047 (12)	−0.00281 (12)	−0.00939 (13)
Mn4	0.01224 (16)	0.01742 (16)	0.01566 (16)	0.00045 (12)	−0.00275 (12)	−0.00872 (13)
O1	0.0193 (8)	0.0177 (8)	0.0293 (9)	0.0016 (6)	−0.0071 (7)	−0.0088 (7)
O2	0.0157 (8)	0.0183 (8)	0.0322 (9)	−0.0017 (6)	−0.0047 (7)	−0.0106 (7)
O3	0.0197 (8)	0.0242 (8)	0.0175 (8)	0.0028 (6)	−0.0042 (6)	−0.0075 (7)
O4	0.0171 (8)	0.0281 (9)	0.0206 (8)	−0.0017 (7)	−0.0013 (6)	−0.0129 (7)
O5	0.0234 (8)	0.0175 (8)	0.0283 (9)	0.0012 (6)	−0.0121 (7)	−0.0069 (7)
O6	0.0197 (8)	0.0154 (7)	0.0243 (8)	−0.0007 (6)	−0.0073 (6)	−0.0078 (6)
O7	0.0248 (9)	0.0285 (9)	0.0197 (8)	0.0076 (7)	−0.0033 (7)	−0.0087 (7)
O8	0.0181 (8)	0.0301 (9)	0.0177 (8)	0.0011 (7)	−0.0040 (6)	−0.0078 (7)
O9	0.0179 (8)	0.0170 (8)	0.0169 (8)	0.0024 (6)	−0.0051 (6)	−0.0084 (7)
O10	0.0223 (8)	0.0192 (8)	0.0252 (8)	−0.0021 (6)	−0.0095 (7)	−0.0093 (7)
O11	0.0180 (8)	0.0212 (8)	0.0196 (8)	−0.0012 (6)	−0.0056 (6)	−0.0108 (6)
O12	0.0158 (8)	0.0331 (9)	0.0180 (8)	−0.0028 (7)	−0.0012 (6)	−0.0117 (7)
O13	0.0181 (8)	0.0220 (8)	0.0177 (8)	0.0013 (6)	−0.0028 (6)	−0.0072 (6)
O14	0.0190 (8)	0.0193 (8)	0.0323 (9)	0.0023 (6)	−0.0055 (7)	−0.0139 (7)
O15	0.0156 (8)	0.0210 (8)	0.0284 (9)	−0.0019 (6)	−0.0032 (6)	−0.0133 (7)
O16	0.0215 (8)	0.0279 (9)	0.0193 (8)	0.0078 (7)	−0.0035 (6)	−0.0061 (7)
O17	0.0189 (8)	0.0281 (9)	0.0181 (8)	0.0040 (7)	−0.0021 (6)	−0.0089 (7)
O18	0.0247 (9)	0.0191 (8)	0.0307 (9)	−0.0009 (7)	−0.0136 (7)	−0.0056 (7)
O19	0.0186 (8)	0.0177 (8)	0.0279 (9)	0.0000 (6)	−0.0067 (6)	−0.0093 (7)
O20	0.0155 (7)	0.0256 (8)	0.0188 (8)	−0.0005 (6)	−0.0053 (6)	−0.0111 (7)
O21	0.0201 (8)	0.0226 (8)	0.0294 (9)	−0.0015 (6)	−0.0091 (7)	−0.0153 (7)
O22	0.0171 (8)	0.0180 (8)	0.0151 (8)	0.0023 (6)	−0.0037 (6)	−0.0081 (6)
N1	0.0189 (9)	0.0196 (9)	0.0170 (9)	0.0013 (7)	−0.0048 (7)	−0.0107 (8)
N2	0.0170 (9)	0.0184 (9)	0.0182 (9)	−0.0009 (7)	−0.0062 (7)	−0.0071 (8)
N3	0.0197 (9)	0.0193 (9)	0.0183 (9)	0.0004 (7)	−0.0038 (7)	−0.0103 (8)
N4	0.0161 (9)	0.0232 (10)	0.0257 (10)	0.0042 (7)	−0.0068 (8)	−0.0155 (8)
N5	0.0156 (9)	0.0197 (9)	0.0169 (9)	0.0011 (7)	−0.0047 (7)	−0.0086 (7)
N6	0.0168 (9)	0.0179 (9)	0.0179 (9)	0.0002 (7)	−0.0033 (7)	−0.0091 (7)
N7	0.0160 (9)	0.0274 (10)	0.0171 (9)	0.0022 (8)	−0.0024 (7)	−0.0115 (8)
N8	0.0173 (9)	0.0200 (9)	0.0223 (10)	0.0030 (7)	−0.0063 (7)	−0.0135 (8)
C1	0.0172 (11)	0.0189 (11)	0.0167 (10)	0.0009 (8)	−0.0019 (8)	−0.0093 (9)
C2	0.0195 (11)	0.0170 (10)	0.0148 (10)	0.0006 (8)	−0.0033 (8)	−0.0062 (9)
C3	0.0170 (11)	0.0205 (11)	0.0185 (11)	0.0001 (9)	−0.0018 (8)	−0.0075 (9)
C4	0.0183 (11)	0.0219 (11)	0.0220 (11)	−0.0048 (9)	−0.0015 (9)	−0.0082 (9)
C5	0.0283 (12)	0.0163 (11)	0.0179 (11)	−0.0035 (9)	−0.0045 (9)	−0.0058 (9)
C6	0.0230 (12)	0.0196 (11)	0.0221 (12)	0.0040 (9)	−0.0056 (9)	−0.0062 (9)
C7	0.0185 (11)	0.0197 (11)	0.0229 (11)	−0.0016 (9)	−0.0048 (9)	−0.0077 (9)
C8	0.0353 (14)	0.0197 (12)	0.0301 (13)	−0.0025 (10)	−0.0080 (11)	−0.0084 (10)
C9	0.0199 (11)	0.0148 (10)	0.0185 (11)	−0.0004 (8)	−0.0028 (9)	−0.0109 (9)
C10	0.0220 (11)	0.0146 (10)	0.0170 (10)	−0.0008 (8)	−0.0035 (9)	−0.0084 (9)
C11	0.0197 (11)	0.0248 (12)	0.0212 (11)	0.0034 (9)	−0.0059 (9)	−0.0105 (10)
C12	0.0327 (13)	0.0243 (12)	0.0225 (12)	0.0068 (10)	−0.0126 (10)	−0.0108 (10)
C13	0.0340 (13)	0.0201 (11)	0.0171 (11)	−0.0031 (10)	−0.0027 (10)	−0.0090 (9)
C14	0.0184 (11)	0.0319 (13)	0.0216 (12)	−0.0036 (10)	−0.0006 (9)	−0.0110 (10)
C15	0.0195 (11)	0.0246 (12)	0.0177 (11)	−0.0003 (9)	−0.0037 (9)	−0.0090 (9)
C16	0.0463 (16)	0.0360 (15)	0.0188 (12)	−0.0075 (12)	−0.0016 (11)	−0.0073 (11)
C17	0.0186 (11)	0.0161 (10)	0.0151 (10)	−0.0006 (8)	−0.0008 (8)	−0.0047 (8)
C18	0.0174 (11)	0.0185 (11)	0.0164 (10)	0.0002 (9)	0.0000 (8)	−0.0061 (9)
C19	0.0270 (13)	0.0223 (12)	0.0368 (14)	0.0034 (10)	−0.0125 (11)	−0.0116 (11)
C20	0.0261 (13)	0.0277 (13)	0.0364 (14)	0.0074 (10)	−0.0114 (11)	−0.0093 (11)
C21	0.0273 (13)	0.0214 (12)	0.0230 (12)	0.0048 (10)	0.0050 (10)	−0.0049 (10)
C22	0.0346 (14)	0.0213 (12)	0.0307 (13)	0.0005 (10)	−0.0039 (11)	−0.0131 (11)
C23	0.0248 (12)	0.0210 (11)	0.0254 (12)	0.0010 (9)	−0.0069 (10)	−0.0092 (10)
C24	0.0328 (14)	0.0240 (13)	0.0397 (15)	0.0092 (11)	0.0024 (12)	−0.0088 (12)
C25	0.0224 (12)	0.0182 (11)	0.0190 (11)	−0.0020 (9)	−0.0046 (9)	−0.0102 (9)
C26	0.0238 (12)	0.0188 (11)	0.0194 (11)	−0.0049 (9)	−0.0063 (9)	−0.0058 (9)
C27	0.0369 (15)	0.0299 (13)	0.0223 (12)	0.0016 (11)	−0.0015 (11)	−0.0100 (11)
C28	0.0489 (18)	0.0406 (16)	0.0227 (13)	−0.0093 (13)	0.0027 (12)	−0.0087 (12)
C29	0.0511 (18)	0.0326 (14)	0.0220 (13)	−0.0196 (13)	−0.0120 (12)	−0.0015 (11)
C30	0.0416 (16)	0.0293 (14)	0.0371 (15)	−0.0065 (12)	−0.0240 (13)	0.0015 (12)
C31	0.0267 (13)	0.0288 (13)	0.0284 (13)	−0.0056 (10)	−0.0084 (10)	−0.0068 (11)
C32	0.088 (3)	0.0488 (19)	0.0237 (15)	−0.0261 (18)	−0.0187 (15)	0.0022 (13)
C33	0.0187 (11)	0.0153 (10)	0.0232 (11)	0.0000 (8)	−0.0066 (9)	−0.0080 (9)
C34	0.0259 (12)	0.0164 (11)	0.0196 (11)	−0.0045 (9)	−0.0017 (9)	−0.0042 (9)
C35	0.0179 (11)	0.0219 (11)	0.0203 (11)	−0.0041 (9)	0.0011 (9)	−0.0101 (9)
C36	0.0149 (10)	0.0180 (10)	0.0183 (11)	−0.0024 (8)	−0.0037 (8)	−0.0117 (9)
C37	0.0179 (11)	0.0173 (10)	0.0155 (10)	−0.0008 (8)	−0.0047 (8)	−0.0077 (9)
C38	0.0173 (11)	0.0193 (11)	0.0201 (11)	−0.0013 (9)	−0.0060 (9)	−0.0047 (9)
C39	0.0238 (12)	0.0149 (10)	0.0243 (12)	0.0008 (9)	−0.0080 (9)	−0.0036 (9)
C40	0.0226 (12)	0.0207 (11)	0.0245 (12)	0.0063 (9)	−0.0087 (9)	−0.0118 (10)
C41	0.0176 (11)	0.0193 (11)	0.0168 (10)	0.0005 (8)	−0.0066 (8)	−0.0101 (9)
C42	0.0192 (11)	0.0156 (10)	0.0160 (10)	0.0006 (8)	−0.0070 (8)	−0.0068 (8)
C43	0.0140 (10)	0.0204 (11)	0.0163 (10)	0.0041 (8)	−0.0058 (8)	−0.0087 (9)
C44	0.0320 (13)	0.0257 (12)	0.0222 (12)	0.0028 (10)	−0.0078 (10)	−0.0169 (10)
C45	0.0306 (14)	0.0350 (14)	0.0400 (15)	0.0027 (11)	−0.0156 (12)	−0.0218 (12)
C46	0.0230 (12)	0.0285 (12)	0.0178 (11)	0.0020 (10)	0.0003 (9)	−0.0114 (10)
C47	0.0297 (13)	0.0298 (13)	0.0243 (12)	−0.0040 (11)	−0.0060 (10)	−0.0053 (11)
C48	0.0135 (10)	0.0199 (11)	0.0149 (10)	−0.0014 (8)	0.0004 (8)	−0.0072 (9)
C49	0.0200 (12)	0.0258 (12)	0.0352 (13)	0.0069 (10)	−0.0102 (10)	−0.0183 (11)
C50	0.0302 (14)	0.0300 (14)	0.0400 (15)	−0.0003 (11)	−0.0114 (12)	−0.0057 (12)
C51	0.0236 (12)	0.0309 (13)	0.0333 (13)	0.0088 (10)	−0.0118 (10)	−0.0257 (11)
C52	0.0260 (13)	0.0370 (15)	0.0519 (17)	0.0011 (11)	−0.0151 (12)	−0.0267 (13)
C53	0.0190 (11)	0.0179 (11)	0.0168 (10)	0.0003 (9)	−0.0021 (8)	−0.0111 (9)
C54	0.0194 (11)	0.0163 (10)	0.0187 (11)	−0.0006 (8)	−0.0044 (8)	−0.0102 (9)
C55	0.0193 (11)	0.0214 (11)	0.0213 (11)	0.0008 (9)	−0.0043 (9)	−0.0080 (9)
C56	0.0195 (12)	0.0270 (12)	0.0254 (12)	−0.0048 (10)	−0.0004 (9)	−0.0103 (10)
C57	0.0339 (14)	0.0190 (11)	0.0200 (11)	−0.0076 (10)	−0.0040 (10)	−0.0086 (9)
C58	0.0296 (13)	0.0197 (11)	0.0235 (12)	0.0037 (10)	−0.0106 (10)	−0.0085 (10)
C59	0.0201 (11)	0.0239 (12)	0.0244 (12)	0.0015 (9)	−0.0052 (9)	−0.0112 (10)
C60	0.0466 (16)	0.0286 (13)	0.0246 (13)	−0.0117 (12)	−0.0068 (11)	−0.0051 (11)
C61	0.0168 (11)	0.0197 (11)	0.0154 (10)	0.0009 (8)	0.0000 (8)	−0.0109 (9)
C62	0.0180 (11)	0.0172 (10)	0.0134 (10)	−0.0001 (8)	−0.0020 (8)	−0.0073 (8)
C63	0.0150 (11)	0.0239 (11)	0.0236 (12)	−0.0016 (9)	−0.0037 (9)	−0.0103 (10)
C64	0.0215 (12)	0.0193 (11)	0.0268 (12)	0.0046 (9)	−0.0061 (9)	−0.0092 (10)
C65	0.0228 (12)	0.0199 (11)	0.0175 (11)	−0.0035 (9)	−0.0030 (9)	−0.0067 (9)
C66	0.0180 (11)	0.0219 (11)	0.0211 (11)	−0.0060 (9)	−0.0038 (9)	−0.0073 (9)
C67	0.0182 (11)	0.0205 (11)	0.0171 (11)	0.0020 (9)	−0.0040 (8)	−0.0072 (9)
C68	0.0296 (13)	0.0200 (12)	0.0337 (14)	−0.0031 (10)	−0.0081 (11)	−0.0075 (10)
C69	0.0189 (11)	0.0180 (11)	0.0189 (11)	−0.0012 (9)	−0.0038 (9)	−0.0087 (9)
C70	0.0237 (12)	0.0177 (11)	0.0168 (11)	−0.0010 (9)	−0.0052 (9)	−0.0067 (9)
C71	0.0212 (12)	0.0216 (11)	0.0263 (12)	0.0016 (9)	−0.0062 (9)	−0.0078 (10)
C72	0.0311 (13)	0.0212 (12)	0.0306 (13)	0.0039 (10)	−0.0163 (11)	−0.0049 (10)
C73	0.0383 (15)	0.0231 (12)	0.0214 (12)	−0.0035 (11)	−0.0109 (11)	−0.0057 (10)
C74	0.0307 (14)	0.0387 (15)	0.0187 (12)	−0.0002 (11)	−0.0013 (10)	−0.0074 (11)
C75	0.0269 (13)	0.0274 (13)	0.0213 (12)	0.0055 (10)	−0.0062 (10)	−0.0064 (10)
C76	0.0464 (17)	0.0319 (14)	0.0253 (13)	−0.0074 (12)	−0.0144 (12)	−0.0009 (11)
C77	0.0182 (11)	0.0186 (11)	0.0171 (11)	−0.0011 (9)	−0.0011 (8)	−0.0053 (9)
C78	0.0223 (12)	0.0179 (11)	0.0196 (11)	0.0002 (9)	−0.0029 (9)	−0.0076 (9)
C79	0.0273 (13)	0.0228 (12)	0.0249 (12)	−0.0016 (10)	−0.0067 (10)	−0.0089 (10)
C80	0.0380 (15)	0.0194 (12)	0.0311 (13)	−0.0035 (10)	−0.0069 (11)	−0.0111 (10)
C81	0.0354 (14)	0.0200 (12)	0.0262 (13)	0.0021 (10)	−0.0011 (11)	−0.0085 (10)
C82	0.0329 (15)	0.0267 (13)	0.0471 (16)	0.0079 (11)	−0.0181 (12)	−0.0107 (12)
C83	0.0296 (13)	0.0205 (12)	0.0397 (15)	0.0007 (10)	−0.0135 (11)	−0.0103 (11)
C84	0.0519 (18)	0.0225 (13)	0.0447 (17)	0.0081 (12)	−0.0090 (14)	−0.0136 (12)
C85	0.0170 (11)	0.0172 (10)	0.0163 (10)	0.0006 (8)	−0.0051 (8)	−0.0093 (9)
C86	0.0136 (10)	0.0228 (11)	0.0160 (10)	0.0022 (8)	−0.0057 (8)	−0.0113 (9)
C87	0.0174 (11)	0.0244 (12)	0.0233 (11)	0.0071 (9)	−0.0074 (9)	−0.0149 (10)
C88	0.0216 (12)	0.0155 (11)	0.0303 (12)	0.0032 (9)	−0.0105 (10)	−0.0101 (10)
C89	0.0174 (11)	0.0186 (11)	0.0215 (11)	−0.0015 (9)	−0.0068 (9)	−0.0070 (9)
C90	0.0186 (11)	0.0170 (11)	0.0217 (11)	0.0016 (9)	−0.0056 (9)	−0.0102 (9)
C91	0.0239 (12)	0.0181 (11)	0.0193 (11)	−0.0030 (9)	−0.0025 (9)	−0.0042 (9)
C92	0.0151 (11)	0.0205 (11)	0.0222 (11)	−0.0043 (9)	0.0014 (9)	−0.0085 (9)
C93	0.0167 (10)	0.0170 (10)	0.0196 (11)	0.0004 (8)	−0.0040 (8)	−0.0133 (9)
C94	0.0170 (11)	0.0170 (10)	0.0159 (10)	−0.0002 (8)	−0.0027 (8)	−0.0081 (9)
C95	0.0119 (10)	0.0218 (11)	0.0176 (10)	0.0045 (8)	−0.0046 (8)	−0.0102 (9)
C96	0.0280 (13)	0.0331 (13)	0.0196 (11)	0.0038 (10)	−0.0052 (9)	−0.0176 (10)
C97	0.0289 (13)	0.0365 (14)	0.0314 (13)	0.0054 (11)	−0.0134 (11)	−0.0203 (12)
C98	0.0191 (12)	0.0404 (14)	0.0173 (11)	−0.0013 (10)	−0.0013 (9)	−0.0101 (10)
C99	0.0274 (13)	0.0345 (14)	0.0287 (13)	−0.0051 (11)	−0.0086 (11)	−0.0024 (11)
C100	0.0110 (10)	0.0210 (11)	0.0152 (10)	−0.0016 (8)	0.0013 (8)	−0.0087 (9)
C101	0.0208 (12)	0.0235 (12)	0.0315 (13)	0.0064 (9)	−0.0113 (10)	−0.0157 (10)
C102	0.0308 (14)	0.0304 (14)	0.0337 (14)	−0.0001 (11)	−0.0121 (11)	−0.0045 (11)
C103	0.0268 (12)	0.0272 (12)	0.0282 (12)	0.0072 (10)	−0.0134 (10)	−0.0217 (10)
C104	0.0266 (13)	0.0288 (13)	0.0458 (15)	0.0027 (10)	−0.0162 (11)	−0.0228 (12)

### Geometric parameters (Å, °)

**Table:**

Mn1—O2	2.1528 (14)	C41—C40	1.393 (3)
Mn1—O3	2.1429 (15)	C41—C42	1.391 (3)
Mn1—O8	2.1743 (15)	C41—C43	1.498 (3)
Mn1—O9	2.1848 (15)	C42—N2	1.336 (3)
Mn1—O10	2.1936 (14)	C42—H42	0.9300
Mn1—N2	2.2854 (18)	C43—O11^ii^	1.246 (2)
Mn2—O1	2.1482 (15)	C44—C45	1.511 (3)
Mn2—O4	2.1197 (15)	C44—H44A	0.9700
Mn2—O6	2.1694 (14)	C44—H44B	0.9700
Mn2—O9	2.2026 (14)	C45—H45A	0.9600
Mn2—O11	2.2109 (13)	C45—H45B	0.9600
Mn2—N1	2.3152 (18)	C45—H45C	0.9600
Mn3—O13	2.1716 (14)	C46—C47	1.512 (3)
Mn3—O15	2.1608 (14)	C46—H46A	0.9700
Mn3—O17	2.1898 (15)	C46—H46B	0.9700
Mn3—O21	2.1755 (14)	C47—H47A	0.9600
Mn3—O22	2.1913 (15)	C47—H47B	0.9600
Mn3—N5	2.2817 (17)	C47—H47C	0.9600
Mn4—O12	2.1071 (15)	C48—O10	1.244 (2)
Mn4—O14	2.1419 (15)	C48—C36^ii^	1.508 (3)
Mn4—O19	2.1540 (15)	C49—C50	1.512 (3)
Mn4—O20	2.1990 (13)	C49—H49A	0.9700
Mn4—O22	2.2069 (14)	C49—H49B	0.9700
Mn4—N6	2.2799 (18)	C50—H50A	0.9600
O1—C1	1.263 (2)	C50—H50B	0.9600
O2—C1	1.252 (2)	C50—H50C	0.9600
O3—C9	1.260 (2)	C51—C52	1.509 (3)
O4—C9	1.258 (2)	C51—H51A	0.9700
O5—C17	1.259 (2)	C51—H51B	0.9700
O6—C17	1.271 (2)	C52—H52A	0.9600
O7—C25	1.257 (3)	C52—H52B	0.9600
O9—H9A	0.94 (3)	C52—H52C	0.9600
O9—H9B	0.937 (18)	C53—C54	1.504 (3)
O11—C43^i^	1.246 (2)	C54—C55	1.383 (3)
O12—C53	1.258 (2)	C54—C59	1.394 (3)
O13—C53	1.261 (2)	C55—H55	0.9300
O14—C61	1.264 (2)	C56—C55	1.382 (3)
O15—C61	1.248 (2)	C56—C57	1.391 (3)
O16—C69	1.260 (2)	C56—H56	0.9300
O17—C69	1.262 (3)	C57—C60	1.505 (3)
O18—C77	1.256 (2)	C58—C59	1.383 (3)
O19—C77	1.263 (2)	C58—C57	1.392 (3)
O22—H22A	0.92 (3)	C58—H58	0.9300
O22—H22B	0.98 (4)	C59—H59	0.9300
N1—C33	1.338 (3)	C60—H60A	0.9600
N1—C37	1.340 (3)	C60—H60B	0.9600
N2—C38	1.343 (3)	C60—H60C	0.9600
N3—C43	1.338 (3)	C62—C61	1.508 (3)
N3—C44	1.473 (3)	C62—C67	1.387 (3)
N3—C46	1.470 (3)	C63—C62	1.392 (3)
N4—C48	1.327 (3)	C63—C64	1.388 (3)
N4—C49	1.472 (3)	C63—H63	0.9300
N4—C51	1.477 (3)	C64—H64	0.9300
N5—C85	1.338 (3)	C65—C66	1.391 (3)
N5—C89	1.338 (3)	C65—C64	1.392 (3)
N6—C90	1.341 (3)	C65—C68	1.509 (3)
N6—C94	1.337 (3)	C66—H66	0.9300
N7—C95	1.331 (3)	C67—C66	1.384 (3)
N7—C96	1.479 (3)	C67—H67	0.9300
N7—C98	1.470 (3)	C68—H68A	0.9600
N8—C100	1.329 (3)	C68—H68B	0.9600
N8—C101	1.474 (3)	C68—H68C	0.9600
N8—C103	1.472 (2)	C70—C69	1.509 (3)
C1—C2	1.508 (3)	C70—C71	1.383 (3)
C3—C2	1.389 (3)	C70—C75	1.391 (3)
C3—H3	0.9300	C71—C72	1.390 (3)
C4—C3	1.383 (3)	C71—H71	0.9300
C4—H4	0.9300	C72—C73	1.382 (3)
C5—C6	1.390 (3)	C72—H72	0.9300
C5—C4	1.391 (3)	C73—C74	1.385 (3)
C5—C8	1.507 (3)	C74—H74	0.9300
C6—C7	1.387 (3)	C75—C74	1.379 (3)
C6—H6	0.9300	C75—H75	0.9300
C7—C2	1.391 (3)	C76—C73	1.514 (3)
C7—H7	0.9300	C76—H76A	0.9600
C8—H8A	0.9600	C76—H76B	0.9600
C8—H8B	0.9600	C76—H76C	0.9600
C8—H8C	0.9600	C77—C78	1.506 (3)
C9—C10	1.496 (3)	C78—C83	1.381 (3)
C10—C15	1.388 (3)	C79—C78	1.384 (3)
C11—C12	1.386 (3)	C79—C80	1.386 (3)
C11—C10	1.394 (3)	C79—H79	0.9300
C11—H11	0.9300	C80—H80	0.9300
C12—C13	1.388 (3)	C81—C80	1.388 (3)
C12—H12	0.9300	C81—C84	1.507 (3)
C14—C13	1.387 (3)	C82—C81	1.389 (3)
C14—C15	1.388 (3)	C82—C83	1.388 (3)
C14—H14	0.9300	C82—H82	0.9300
C15—H15	0.9300	C83—H83	0.9300
C16—C13	1.505 (3)	C84—H84A	0.9600
C16—H16A	0.9600	C84—H84B	0.9600
C16—H16B	0.9600	C84—H84C	0.9600
C16—H16C	0.9600	C85—H85	0.9300
C18—C17	1.498 (3)	C86—C85	1.389 (3)
C18—C19	1.384 (3)	C86—C95^i^	1.497 (3)
C18—C23	1.389 (3)	C87—C88	1.382 (3)
C19—H19	0.9300	C87—C86	1.395 (3)
C20—C21	1.385 (3)	C87—H87	0.9300
C20—C19	1.387 (3)	C88—H88	0.9300
C20—H20	0.9300	C89—C88	1.376 (3)
C21—C22	1.391 (3)	C89—H89	0.9300
C21—C24	1.504 (3)	C90—H90	0.9300
C22—C23	1.383 (3)	C91—C90	1.380 (3)
C22—H22	0.9300	C91—C92	1.381 (3)
C23—H23	0.9300	C91—H91	0.9300
C24—H24A	0.9600	C92—H92	0.9300
C24—H24B	0.9600	C93—C92	1.389 (3)
C24—H24C	0.9600	C93—C94	1.386 (3)
C25—O8	1.267 (3)	C93—C100^ii^	1.502 (3)
C25—C26	1.506 (3)	C94—H94	0.9300
C26—C27	1.393 (3)	C95—O20	1.243 (2)
C26—C31	1.379 (3)	C95—C86^ii^	1.497 (3)
C27—H27	0.9300	C96—C97	1.516 (3)
C28—C27	1.380 (3)	C96—H96A	0.9700
C28—H28	0.9300	C96—H96B	0.9700
C29—C28	1.388 (4)	C97—H97A	0.9600
C29—C30	1.380 (4)	C97—H97B	0.9600
C30—H30	0.9300	C97—H97C	0.9600
C31—C30	1.394 (3)	C98—C99	1.517 (3)
C31—H31	0.9300	C98—H98A	0.9700
C32—C29	1.507 (3)	C98—H98B	0.9700
C32—H32A	0.9600	C99—H99A	0.9600
C32—H32B	0.9600	C99—H99B	0.9600
C32—H32C	0.9600	C99—H99C	0.9600
C33—C34	1.383 (3)	C100—O21	1.243 (2)
C33—H33	0.9300	C100—C93^i^	1.502 (3)
C34—H34	0.9300	C101—C102	1.509 (3)
C35—C34	1.380 (3)	C101—H10A	0.9700
C35—C36	1.387 (3)	C101—H10B	0.9700
C35—H35	0.9300	C102—H10H	0.9600
C36—C48^i^	1.508 (3)	C102—H10I	0.9600
C37—C36	1.387 (3)	C102—H10J	0.9600
C37—H37	0.9300	C103—C104	1.512 (3)
C38—H38	0.9300	C103—H10C	0.9700
C39—C38	1.383 (3)	C103—H10D	0.9700
C39—C40	1.380 (3)	C104—H10E	0.9600
C39—H39	0.9300	C104—H10F	0.9600
C40—H40	0.9300	C104—H10G	0.9600
			
O2—Mn1—O8	86.85 (6)	N2—C42—H42	118.1
O2—Mn1—O9	92.31 (6)	C41—C42—H42	118.1
O2—Mn1—O10	174.70 (6)	O11^ii^—C43—N3	121.49 (19)
O2—Mn1—N2	84.43 (6)	O11^ii^—C43—C41	118.48 (18)
O3—Mn1—O2	97.60 (6)	N3—C43—C41	120.00 (18)
O3—Mn1—O8	175.43 (6)	N3—C44—C45	113.85 (19)
O3—Mn1—O9	91.91 (6)	N3—C44—H44A	108.8
O3—Mn1—O10	86.29 (6)	N3—C44—H44B	108.8
O3—Mn1—N2	85.17 (6)	C45—C44—H44A	108.8
O8—Mn1—O9	88.92 (6)	C45—C44—H44B	108.8
O8—Mn1—O10	89.21 (6)	H44A—C44—H44B	107.7
O8—Mn1—N2	94.29 (6)	C44—C45—H45A	109.5
O9—Mn1—O10	91.16 (6)	C44—C45—H45B	109.5
O9—Mn1—N2	175.29 (6)	C44—C45—H45C	109.5
O10—Mn1—N2	92.34 (6)	H45A—C45—H45B	109.5
O1—Mn2—O6	170.61 (6)	H45A—C45—H45C	109.5
O1—Mn2—O9	89.49 (6)	H45B—C45—H45C	109.5
O1—Mn2—O11	88.90 (5)	N3—C46—C47	113.19 (18)
O1—Mn2—N1	86.27 (6)	N3—C46—H46A	108.9
O4—Mn2—O1	97.75 (6)	N3—C46—H46B	108.9
O4—Mn2—O6	91.45 (6)	C47—C46—H46A	108.9
O4—Mn2—O9	90.02 (6)	C47—C46—H46B	108.9
O4—Mn2—O11	89.10 (5)	H46A—C46—H46B	107.8
O4—Mn2—N1	174.27 (6)	C46—C47—H47A	109.5
O6—Mn2—O9	88.61 (5)	C46—C47—H47B	109.5
O6—Mn2—O11	93.15 (5)	C46—C47—H47C	109.5
O6—Mn2—N1	84.69 (6)	H47A—C47—H47B	109.5
O9—Mn2—O11	178.04 (6)	H47A—C47—H47C	109.5
O9—Mn2—N1	94.11 (6)	H47B—C47—H47C	109.5
O11—Mn2—N1	86.89 (6)	O10—C48—N4	121.96 (19)
O13—Mn3—O17	170.86 (6)	O10—C48—C36^ii^	119.09 (18)
O13—Mn3—O21	84.47 (6)	N4—C48—C36^ii^	118.91 (17)
O13—Mn3—O22	89.52 (6)	N4—C49—C50	113.82 (19)
O13—Mn3—N5	83.28 (6)	N4—C49—H49A	108.8
O15—Mn3—O13	100.63 (6)	N4—C49—H49B	108.8
O15—Mn3—O17	88.49 (6)	C50—C49—H49A	108.8
O15—Mn3—O21	172.45 (6)	C50—C49—H49B	108.8
O15—Mn3—O22	95.02 (6)	H49A—C49—H49B	107.7
O15—Mn3—N5	84.32 (6)	C49—C50—H50A	109.5
O17—Mn3—O22	89.03 (6)	C49—C50—H50B	109.5
O17—Mn3—N5	98.40 (6)	C49—C50—H50C	109.5
O21—Mn3—O17	86.52 (6)	H50A—C50—H50B	109.5
O21—Mn3—O22	90.55 (6)	H50A—C50—H50C	109.5
O21—Mn3—N5	90.82 (6)	H50B—C50—H50C	109.5
O22—Mn3—N5	172.51 (6)	N4—C51—C52	113.39 (19)
O12—Mn4—O14	97.04 (6)	N4—C51—H51A	108.9
O12—Mn4—O19	90.47 (6)	N4—C51—H51B	108.9
O12—Mn4—O20	91.25 (5)	C52—C51—H51A	108.9
O12—Mn4—O22	88.94 (6)	C52—C51—H51B	108.9
O12—Mn4—N6	175.55 (6)	H51A—C51—H51B	107.7
O14—Mn4—O19	172.28 (6)	C51—C52—H52A	109.5
O14—Mn4—O20	89.45 (6)	C51—C52—H52B	109.5
O14—Mn4—O22	92.31 (6)	C51—C52—H52C	109.5
O14—Mn4—N6	86.78 (6)	H52A—C52—H52B	109.5
O19—Mn4—O20	88.57 (5)	H52A—C52—H52C	109.5
O19—Mn4—O22	89.64 (6)	H52B—C52—H52C	109.5
O19—Mn4—N6	85.65 (6)	O12—C53—O13	125.68 (19)
O20—Mn4—O22	178.20 (6)	O12—C53—C54	116.39 (18)
O20—Mn4—N6	86.48 (6)	O13—C53—C54	117.92 (18)
O22—Mn4—N6	93.20 (6)	C55—C54—C53	121.96 (19)
C1—O1—Mn2	136.20 (13)	C55—C54—C59	118.4 (2)
C1—O2—Mn1	135.80 (14)	C59—C54—C53	119.61 (19)
C9—O3—Mn1	132.79 (14)	C54—C55—H55	119.7
C9—O4—Mn2	139.38 (13)	C56—C55—C54	120.7 (2)
C17—O6—Mn2	126.66 (13)	C56—C55—H55	119.7
C25—O8—Mn1	126.00 (13)	C55—C56—C57	121.5 (2)
Mn1—O9—Mn2	114.68 (6)	C55—C56—H56	119.2
Mn1—O9—H9A	96.9 (17)	C57—C56—H56	119.2
Mn1—O9—H9B	120 (2)	C56—C57—C58	117.5 (2)
Mn2—O9—H9A	118.6 (17)	C56—C57—C60	121.4 (2)
Mn2—O9—H9B	95 (2)	C58—C57—C60	121.1 (2)
H9A—O9—H9B	113 (3)	C57—C58—H58	119.4
C48—O10—Mn1	139.39 (14)	C59—C58—C57	121.2 (2)
C43^i^—O11—Mn2	144.18 (14)	C59—C58—H58	119.4
C53—O12—Mn4	138.85 (13)	C54—C59—H59	119.7
C53—O13—Mn3	131.60 (14)	C58—C59—C54	120.6 (2)
C61—O14—Mn4	137.28 (13)	C58—C59—H59	119.7
C61—O15—Mn3	134.89 (14)	C57—C60—H60A	109.5
C69—O17—Mn3	126.80 (13)	C57—C60—H60B	109.5
C77—O19—Mn4	125.56 (13)	C57—C60—H60C	109.5
C95—O20—Mn4	143.45 (14)	H60A—C60—H60B	109.5
C100—O21—Mn3	146.13 (14)	H60A—C60—H60C	109.5
Mn3—O22—Mn4	113.16 (6)	H60B—C60—H60C	109.5
Mn3—O22—H22A	99.9 (16)	O14—C61—C62	115.51 (18)
Mn3—O22—H22B	119 (2)	O15—C61—O14	126.27 (19)
Mn4—O22—H22A	118.4 (16)	O15—C61—C62	118.21 (18)
Mn4—O22—H22B	95 (2)	C63—C62—C61	121.92 (18)
H22A—O22—H22B	113 (3)	C67—C62—C61	119.52 (18)
C33—N1—Mn2	124.49 (14)	C67—C62—C63	118.56 (19)
C33—N1—C37	117.62 (18)	C62—C63—H63	119.8
C37—N1—Mn2	117.75 (14)	C64—C63—C62	120.5 (2)
C38—N2—Mn1	121.24 (14)	C64—C63—H63	119.8
C42—N2—Mn1	120.29 (13)	C63—C64—C65	121.0 (2)
C42—N2—C38	117.38 (18)	C63—C64—H64	119.5
C43—N3—C44	124.34 (18)	C65—C64—H64	119.5
C43—N3—C46	117.55 (17)	C64—C65—C68	121.5 (2)
C46—N3—C44	117.64 (17)	C66—C65—C64	118.06 (19)
C48—N4—C49	119.05 (17)	C66—C65—C68	120.40 (19)
C48—N4—C51	122.86 (18)	C65—C66—H66	119.5
C49—N4—C51	118.08 (17)	C67—C66—C65	121.0 (2)
C85—N5—Mn3	118.94 (13)	C67—C66—H66	119.5
C85—N5—C89	117.71 (18)	C62—C67—H67	119.6
C89—N5—Mn3	122.73 (14)	C66—C67—C62	120.9 (2)
C90—N6—Mn4	126.99 (14)	C66—C67—H67	119.6
C94—N6—Mn4	115.46 (14)	C65—C68—H68A	109.5
C94—N6—C90	117.40 (18)	C65—C68—H68B	109.5
C95—N7—C96	123.95 (18)	C65—C68—H68C	109.5
C95—N7—C98	117.56 (18)	H68A—C68—H68B	109.5
C98—N7—C96	117.96 (17)	H68A—C68—H68C	109.5
C100—N8—C101	118.32 (17)	H68B—C68—H68C	109.5
C100—N8—C103	123.07 (17)	O16—C69—O17	125.0 (2)
C103—N8—C101	118.50 (17)	O16—C69—C70	116.66 (19)
O1—C1—C2	116.18 (18)	O17—C69—C70	118.28 (19)
O2—C1—O1	126.35 (19)	C71—C70—C69	121.2 (2)
O2—C1—C2	117.45 (18)	C71—C70—C75	118.2 (2)
C3—C2—C1	119.81 (19)	C75—C70—C69	120.65 (19)
C3—C2—C7	118.72 (19)	C70—C71—C72	120.3 (2)
C7—C2—C1	121.42 (18)	C70—C71—H71	119.8
C2—C3—H3	119.7	C72—C71—H71	119.8
C4—C3—C2	120.7 (2)	C71—C72—H72	119.2
C4—C3—H3	119.7	C73—C72—C71	121.7 (2)
C3—C4—C5	120.9 (2)	C73—C72—H72	119.2
C3—C4—H4	119.5	C72—C73—C74	117.5 (2)
C5—C4—H4	119.5	C72—C73—C76	122.1 (2)
C4—C5—C8	120.3 (2)	C74—C73—C76	120.3 (2)
C6—C5—C4	118.21 (19)	C73—C74—H74	119.3
C6—C5—C8	121.4 (2)	C75—C74—C73	121.4 (2)
C5—C6—H6	119.5	C75—C74—H74	119.3
C7—C6—C5	121.0 (2)	C70—C75—H75	119.5
C7—C6—H6	119.5	C74—C75—C70	120.9 (2)
C2—C7—H7	119.8	C74—C75—H75	119.5
C6—C7—C2	120.3 (2)	C73—C76—H76A	109.5
C6—C7—H7	119.8	C73—C76—H76B	109.5
C5—C8—H8A	109.5	C73—C76—H76C	109.5
C5—C8—H8B	109.5	H76A—C76—H76B	109.5
C5—C8—H8C	109.5	H76A—C76—H76C	109.5
H8A—C8—H8B	109.5	H76B—C76—H76C	109.5
H8A—C8—H8C	109.5	O18—C77—O19	125.1 (2)
H8B—C8—H8C	109.5	O18—C77—C78	117.55 (19)
O3—C9—C10	117.14 (18)	O19—C77—C78	117.31 (18)
O4—C9—O3	125.93 (19)	C83—C78—C79	118.6 (2)
O4—C9—C10	116.90 (18)	C83—C78—C77	121.03 (19)
C11—C10—C9	120.30 (19)	C79—C78—C77	120.38 (19)
C15—C10—C9	121.41 (19)	C78—C79—C80	120.6 (2)
C15—C10—C11	118.3 (2)	C78—C79—H79	119.7
C10—C11—H11	119.7	C80—C79—H79	119.7
C12—C11—C10	120.5 (2)	C79—C80—C81	121.4 (2)
C12—C11—H11	119.7	C79—C80—H80	119.3
C11—C12—C13	121.1 (2)	C81—C80—H80	119.3
C11—C12—H12	119.4	C80—C81—C82	117.5 (2)
C13—C12—H12	119.4	C80—C81—C84	121.5 (2)
C12—C13—C16	120.8 (2)	C82—C81—C84	121.0 (2)
C14—C13—C12	118.3 (2)	C81—C82—H82	119.3
C14—C13—C16	120.9 (2)	C83—C82—C81	121.3 (2)
C13—C14—C15	120.7 (2)	C83—C82—H82	119.3
C13—C14—H14	119.6	C78—C83—C82	120.6 (2)
C15—C14—H14	119.6	C78—C83—H83	119.7
C10—C15—C14	121.0 (2)	C82—C83—H83	119.7
C10—C15—H15	119.5	C81—C84—H84A	109.5
C14—C15—H15	119.5	C81—C84—H84B	109.5
C13—C16—H16A	109.5	C81—C84—H84C	109.5
C13—C16—H16B	109.5	H84A—C84—H84B	109.5
C13—C16—H16C	109.5	H84A—C84—H84C	109.5
H16A—C16—H16B	109.5	H84B—C84—H84C	109.5
H16A—C16—H16C	109.5	N5—C85—C86	123.48 (19)
H16B—C16—H16C	109.5	N5—C85—H85	118.3
O5—C17—O6	124.73 (19)	C86—C85—H85	118.3
O5—C17—C18	117.28 (18)	C85—C86—C87	117.8 (2)
O6—C17—C18	117.99 (18)	C85—C86—C95^i^	117.21 (18)
C19—C18—C17	121.26 (19)	C87—C86—C95^i^	124.91 (19)
C19—C18—C23	118.5 (2)	C88—C87—C86	118.8 (2)
C23—C18—C17	120.26 (19)	C88—C87—H87	120.6
C18—C19—C20	120.7 (2)	C86—C87—H87	120.6
C18—C19—H19	119.6	C87—C88—H88	120.4
C20—C19—H19	119.6	C89—C88—C87	119.3 (2)
C19—C20—H20	119.4	C89—C88—H88	120.4
C21—C20—C19	121.2 (2)	N5—C89—C88	122.9 (2)
C21—C20—H20	119.4	N5—C89—H89	118.5
C20—C21—C22	117.8 (2)	C88—C89—H89	118.5
C20—C21—C24	121.6 (2)	N6—C90—C91	122.7 (2)
C22—C21—C24	120.6 (2)	N6—C90—H90	118.7
C21—C22—H22	119.4	C91—C90—H90	118.7
C23—C22—C21	121.3 (2)	C90—C91—C92	119.6 (2)
C23—C22—H22	119.4	C90—C91—H91	120.2
C18—C23—H23	119.7	C92—C91—H91	120.2
C22—C23—C18	120.5 (2)	C91—C92—C93	118.3 (2)
C22—C23—H23	119.7	C91—C92—H92	120.8
C21—C24—H24A	109.5	C93—C92—H92	120.8
C21—C24—H24B	109.5	C92—C93—C100^ii^	124.11 (19)
C21—C24—H24C	109.5	C94—C93—C92	118.32 (19)
H24A—C24—H24B	109.5	C94—C93—C100^ii^	117.50 (18)
H24A—C24—H24C	109.5	N6—C94—C93	123.62 (19)
H24B—C24—H24C	109.5	N6—C94—H94	118.2
O7—C25—O8	125.4 (2)	C93—C94—H94	118.2
O7—C25—C26	116.86 (19)	O20—C95—N7	121.7 (2)
O8—C25—C26	117.77 (19)	O20—C95—C86^ii^	118.05 (18)
C31—C26—C27	118.6 (2)	N7—C95—C86^ii^	120.13 (18)
C31—C26—C25	120.8 (2)	N7—C96—C97	113.40 (19)
C27—C26—C25	120.5 (2)	N7—C96—H96A	108.9
C26—C27—H27	119.8	N7—C96—H96B	108.9
C28—C27—C26	120.4 (2)	C97—C96—H96A	108.9
C28—C27—H27	119.8	C97—C96—H96B	108.9
C27—C28—C29	121.5 (3)	H96A—C96—H96B	107.7
C27—C28—H28	119.2	C96—C97—H97A	109.5
C29—C28—H28	119.2	C96—C97—H97B	109.5
C28—C29—C32	120.7 (3)	C96—C97—H97C	109.5
C30—C29—C28	117.6 (2)	H97A—C97—H97B	109.5
C30—C29—C32	121.7 (3)	H97A—C97—H97C	109.5
C29—C30—C31	121.5 (3)	H97B—C97—H97C	109.5
C29—C30—H30	119.3	N7—C98—C99	113.18 (19)
C31—C30—H30	119.3	N7—C98—H98A	108.9
C26—C31—C30	120.3 (2)	N7—C98—H98B	108.9
C26—C31—H31	119.8	C99—C98—H98A	108.9
C30—C31—H31	119.8	C99—C98—H98B	108.9
C29—C32—H32A	109.5	H98A—C98—H98B	107.8
C29—C32—H32B	109.5	C98—C99—H99A	109.5
C29—C32—H32C	109.5	C98—C99—H99B	109.5
H32A—C32—H32B	109.5	C98—C99—H99C	109.5
H32A—C32—H32C	109.5	H99A—C99—H99B	109.5
H32B—C32—H32C	109.5	H99A—C99—H99C	109.5
N1—C33—C34	122.7 (2)	H99B—C99—H99C	109.5
N1—C33—H33	118.7	O21—C100—N8	121.72 (19)
C34—C33—H33	118.7	O21—C100—C93^i^	119.68 (18)
C33—C34—H34	120.3	N8—C100—C93^i^	118.45 (17)
C35—C34—C33	119.4 (2)	N8—C101—C102	112.10 (19)
C35—C34—H34	120.3	N8—C101—H10A	109.2
C34—C35—C36	118.6 (2)	N8—C101—H10B	109.2
C34—C35—H35	120.7	C102—C101—H10A	109.2
C36—C35—H35	120.7	C102—C101—H10B	109.2
C35—C36—C37	118.39 (19)	H10A—C101—H10B	107.9
C35—C36—C48^i^	122.52 (19)	C101—C102—H10H	109.5
C37—C36—C48^i^	119.01 (18)	C101—C102—H10I	109.5
N1—C37—C36	123.30 (19)	C101—C102—H10J	109.5
N1—C37—H37	118.3	H10H—C102—H10I	109.5
C36—C37—H37	118.3	H10H—C102—H10J	109.5
N2—C38—C39	123.0 (2)	H10I—C102—H10J	109.5
N2—C38—H38	118.5	N8—C103—C104	113.38 (18)
C39—C38—H38	118.5	N8—C103—H10C	108.9
C38—C39—H39	120.5	N8—C103—H10D	108.9
C40—C39—C38	119.0 (2)	C104—C103—H10C	108.9
C40—C39—H39	120.5	C104—C103—H10D	108.9
C39—C40—C41	119.1 (2)	H10C—C103—H10D	107.7
C39—C40—H40	120.5	C103—C104—H10E	109.5
C41—C40—H40	120.5	C103—C104—H10F	109.5
C40—C41—C43	124.26 (19)	C103—C104—H10G	109.5
C42—C41—C40	117.7 (2)	H10E—C104—H10F	109.5
C42—C41—C43	117.84 (18)	H10E—C104—H10G	109.5
N2—C42—C41	123.75 (19)	H10F—C104—H10G	109.5
			
O3—Mn1—O2—C1	−79.9 (2)	C96—N7—C95—C86^ii^	−3.1 (3)
O8—Mn1—O2—C1	101.1 (2)	C98—N7—C95—O20	1.9 (3)
O9—Mn1—O2—C1	12.3 (2)	C98—N7—C95—C86^ii^	−174.63 (18)
N2—Mn1—O2—C1	−164.3 (2)	C95—N7—C96—C97	−83.4 (3)
O2—Mn1—O3—C9	66.64 (18)	C98—N7—C96—C97	88.1 (2)
O9—Mn1—O3—C9	−25.94 (18)	C95—N7—C98—C99	72.9 (2)
O10—Mn1—O3—C9	−116.98 (18)	C96—N7—C98—C99	−99.1 (2)
N2—Mn1—O3—C9	150.36 (18)	C101—N8—C100—O21	3.8 (3)
O2—Mn1—O8—C25	−73.48 (17)	C101—N8—C100—C93^i^	−171.83 (18)
O9—Mn1—O8—C25	18.89 (17)	C103—N8—C100—O21	179.7 (2)
O10—Mn1—O8—C25	110.06 (17)	C103—N8—C100—C93^i^	4.2 (3)
N2—Mn1—O8—C25	−157.65 (17)	C100—N8—C101—C102	72.1 (2)
O3—Mn1—O9—Mn2	51.35 (8)	C103—N8—C101—C102	−104.1 (2)
O2—Mn1—O9—Mn2	−46.34 (8)	C100—N8—C103—C104	−98.1 (2)
O8—Mn1—O9—Mn2	−133.14 (8)	C101—N8—C103—C104	77.9 (2)
O10—Mn1—O9—Mn2	137.67 (7)	O1—C1—C2—C3	−11.1 (3)
O3—Mn1—O10—C48	−152.6 (2)	O1—C1—C2—C7	166.19 (19)
O8—Mn1—O10—C48	26.7 (2)	O2—C1—C2—C3	170.53 (19)
O9—Mn1—O10—C48	115.6 (2)	O2—C1—C2—C7	−12.2 (3)
N2—Mn1—O10—C48	−67.6 (2)	C4—C3—C2—C1	174.29 (19)
O2—Mn1—N2—C38	−29.95 (15)	C4—C3—C2—C7	−3.1 (3)
O2—Mn1—N2—C42	137.82 (15)	C5—C4—C3—C2	−0.2 (3)
O3—Mn1—N2—C38	−128.09 (15)	C6—C5—C4—C3	3.5 (3)
O3—Mn1—N2—C42	39.68 (14)	C8—C5—C4—C3	−172.6 (2)
O8—Mn1—N2—C38	56.46 (15)	C4—C5—C6—C7	−3.4 (3)
O8—Mn1—N2—C42	−135.77 (14)	C8—C5—C6—C7	172.6 (2)
O10—Mn1—N2—C38	145.84 (15)	C5—C6—C7—C2	0.1 (3)
O10—Mn1—N2—C42	−46.39 (15)	C6—C7—C2—C1	−174.18 (19)
O4—Mn2—O1—C1	56.3 (2)	C6—C7—C2—C3	3.1 (3)
O9—Mn2—O1—C1	−33.7 (2)	O4—C9—C10—C15	−169.21 (19)
O11—Mn2—O1—C1	145.2 (2)	O3—C9—C10—C15	9.3 (3)
N1—Mn2—O1—C1	−127.8 (2)	O4—C9—C10—C11	11.9 (3)
O1—Mn2—O4—C9	−79.6 (2)	O3—C9—C10—C11	−169.58 (19)
O6—Mn2—O4—C9	98.5 (2)	C11—C10—C15—C14	0.1 (3)
O9—Mn2—O4—C9	9.9 (2)	C9—C10—C15—C14	−178.75 (19)
O11—Mn2—O4—C9	−168.3 (2)	C12—C11—C10—C15	−0.9 (3)
O4—Mn2—O6—C17	−79.50 (17)	C12—C11—C10—C9	177.93 (19)
O9—Mn2—O6—C17	10.48 (17)	C10—C11—C12—C13	0.5 (3)
O11—Mn2—O6—C17	−168.68 (17)	C15—C14—C13—C12	−1.5 (3)
N1—Mn2—O6—C17	104.74 (17)	C15—C14—C13—C16	178.7 (2)
O1—Mn2—O9—Mn1	51.85 (8)	C11—C12—C13—C14	0.7 (3)
O4—Mn2—O9—Mn1	−45.90 (8)	C11—C12—C13—C16	−179.5 (2)
O6—Mn2—O9—Mn1	−137.35 (8)	C13—C14—C15—C10	1.1 (3)
N1—Mn2—O9—Mn1	138.08 (8)	C19—C18—C17—O5	172.8 (2)
O1—Mn2—N1—C33	30.76 (16)	C19—C18—C17—O6	−8.2 (3)
O1—Mn2—N1—C37	−144.83 (14)	C23—C18—C17—O5	−6.7 (3)
O6—Mn2—N1—C33	−146.69 (16)	C23—C18—C17—O6	172.3 (2)
O6—Mn2—N1—C37	37.72 (14)	C17—C18—C19—C20	179.9 (2)
O9—Mn2—N1—C33	−58.46 (16)	C23—C18—C19—C20	−0.6 (4)
O9—Mn2—N1—C37	125.94 (14)	C17—C18—C23—C22	179.8 (2)
O11—Mn2—N1—C33	119.86 (16)	C19—C18—C23—C22	0.3 (3)
O11—Mn2—N1—C37	−55.74 (14)	C21—C20—C19—C18	0.0 (4)
O15—Mn3—O13—C53	−66.18 (18)	C19—C20—C21—C22	0.9 (4)
O21—Mn3—O13—C53	119.44 (18)	C19—C20—C21—C24	179.8 (2)
O22—Mn3—O13—C53	28.84 (18)	C20—C21—C22—C23	−1.1 (4)
N5—Mn3—O13—C53	−149.08 (18)	C24—C21—C22—C23	180.0 (2)
O13—Mn3—O15—C61	74.1 (2)	C21—C22—C23—C18	0.5 (4)
O17—Mn3—O15—C61	−105.2 (2)	O7—C25—O8—Mn1	−19.4 (3)
O22—Mn3—O15—C61	−16.3 (2)	C26—C25—O8—Mn1	160.32 (13)
N5—Mn3—O15—C61	156.2 (2)	O7—C25—C26—C27	−5.1 (3)
O15—Mn3—O17—C69	83.06 (17)	O7—C25—C26—C31	171.9 (2)
O21—Mn3—O17—C69	−102.60 (17)	O8—C25—C26—C27	175.1 (2)
O22—Mn3—O17—C69	−11.99 (17)	O8—C25—C26—C31	−7.9 (3)
N5—Mn3—O17—C69	167.09 (17)	C31—C26—C27—C28	0.5 (3)
O13—Mn3—O21—C100	156.8 (3)	C25—C26—C27—C28	177.6 (2)
O17—Mn3—O21—C100	−24.7 (3)	C25—C26—C31—C30	−176.5 (2)
O22—Mn3—O21—C100	−113.7 (3)	C27—C26—C31—C30	0.6 (3)
N5—Mn3—O21—C100	73.6 (3)	C29—C28—C27—C26	−1.3 (4)
O13—Mn3—O22—Mn4	−56.91 (8)	C30—C29—C28—C27	1.1 (4)
O15—Mn3—O22—Mn4	43.72 (8)	C32—C29—C28—C27	−178.4 (2)
O17—Mn3—O22—Mn4	132.11 (8)	C28—C29—C30—C31	0.0 (4)
O21—Mn3—O22—Mn4	−141.38 (8)	C32—C29—C30—C31	179.5 (2)
O13—Mn3—N5—C85	−44.99 (14)	C26—C31—C30—C29	−0.8 (4)
O13—Mn3—N5—C89	125.77 (16)	N1—C33—C34—C35	0.8 (3)
O15—Mn3—N5—C85	−146.43 (14)	C36—C35—C34—C33	−1.5 (3)
O15—Mn3—N5—C89	24.33 (15)	C34—C35—C36—C37	0.8 (3)
O17—Mn3—N5—C85	125.95 (14)	C34—C35—C36—C48^i^	177.45 (18)
O17—Mn3—N5—C89	−63.30 (16)	N1—C37—C36—C35	0.8 (3)
O21—Mn3—N5—C85	39.35 (14)	N1—C37—C36—C48^i^	−175.99 (18)
O21—Mn3—N5—C89	−149.90 (15)	C40—C39—C38—N2	0.0 (3)
O14—Mn4—O12—C53	84.7 (2)	C38—C39—C40—C41	1.6 (3)
O19—Mn4—O12—C53	−97.1 (2)	C42—C41—C40—C39	−1.5 (3)
O20—Mn4—O12—C53	174.3 (2)	C43—C41—C40—C39	173.70 (18)
O22—Mn4—O12—C53	−7.5 (2)	C40—C41—C42—N2	−0.2 (3)
O12—Mn4—O14—C61	−59.5 (2)	C43—C41—C42—N2	−175.73 (17)
O20—Mn4—O14—C61	−150.7 (2)	C40—C41—C43—O11^ii^	−120.2 (2)
O22—Mn4—O14—C61	29.7 (2)	C40—C41—C43—N3	61.7 (3)
N6—Mn4—O14—C61	122.8 (2)	C42—C41—C43—O11^ii^	55.0 (3)
O12—Mn4—O19—C77	66.94 (17)	C42—C41—C43—N3	−123.1 (2)
O20—Mn4—O19—C77	158.19 (17)	C41—C42—N2—Mn1	−166.44 (15)
O22—Mn4—O19—C77	−22.00 (17)	C41—C42—N2—C38	1.8 (3)
N6—Mn4—O19—C77	−115.24 (17)	N4—C48—O10—Mn1	−132.5 (2)
O12—Mn4—O20—C95	−86.5 (2)	C36^ii^—C48—O10—Mn1	49.8 (3)
O14—Mn4—O20—C95	10.5 (2)	O12—C53—C54—C55	167.60 (19)
O19—Mn4—O20—C95	−177.0 (2)	O12—C53—C54—C59	−11.5 (3)
N6—Mn4—O20—C95	97.3 (2)	O13—C53—C54—C55	−10.8 (3)
O12—Mn4—O22—Mn3	50.24 (8)	O13—C53—C54—C59	170.05 (19)
O14—Mn4—O22—Mn3	−46.76 (8)	C53—C54—C55—C56	−177.29 (19)
O19—Mn4—O22—Mn3	140.72 (8)	C59—C54—C55—C56	1.8 (3)
N6—Mn4—O22—Mn3	−133.66 (8)	C55—C54—C59—C58	−1.1 (3)
O14—Mn4—N6—C90	−37.95 (16)	C53—C54—C59—C58	178.02 (19)
O14—Mn4—N6—C94	137.43 (14)	C57—C56—C55—C54	−0.9 (3)
O19—Mn4—N6—C90	143.58 (17)	C55—C56—C57—C58	−0.8 (3)
O19—Mn4—N6—C94	−41.05 (14)	C55—C56—C57—C60	178.4 (2)
O20—Mn4—N6—C90	−127.59 (17)	C59—C58—C57—C56	1.6 (3)
O20—Mn4—N6—C94	47.78 (14)	C59—C58—C57—C60	−177.7 (2)
O22—Mn4—N6—C90	54.18 (17)	C57—C58—C59—C54	−0.6 (3)
O22—Mn4—N6—C94	−130.44 (14)	C67—C62—C61—O14	12.2 (3)
Mn2—O1—C1—O2	1.6 (4)	C67—C62—C61—O15	−168.26 (19)
Mn2—O1—C1—C2	−176.61 (13)	C63—C62—C61—O14	−168.89 (19)
Mn1—O2—C1—O1	12.9 (3)	C63—C62—C61—O15	10.7 (3)
Mn1—O2—C1—C2	−168.92 (14)	C61—C62—C67—C66	−179.78 (19)
Mn1—O3—C9—O4	−9.7 (3)	C63—C62—C67—C66	1.3 (3)
Mn1—O3—C9—C10	172.00 (13)	C64—C63—C62—C61	−179.99 (19)
Mn2—O4—C9—O3	21.0 (3)	C64—C63—C62—C67	−1.1 (3)
Mn2—O4—C9—C10	−160.69 (15)	C62—C63—C64—C65	0.0 (3)
Mn2—O6—C17—O5	3.8 (3)	C66—C65—C64—C63	0.8 (3)
Mn2—O6—C17—C18	−175.16 (13)	C68—C65—C64—C63	−177.7 (2)
C43^i^—O11—Mn2—O1	0.3 (2)	C64—C65—C66—C67	−0.6 (3)
C43^i^—O11—Mn2—O4	98.0 (2)	C68—C65—C66—C67	177.9 (2)
C43^i^—O11—Mn2—O6	−170.5 (2)	C62—C67—C66—C65	−0.4 (3)
C43^i^—O11—Mn2—N1	−86.0 (2)	C71—C70—C69—O16	175.77 (19)
Mn4—O12—C53—O13	−27.6 (3)	C71—C70—C69—O17	−5.0 (3)
Mn4—O12—C53—C54	154.13 (16)	C75—C70—C69—O16	−6.0 (3)
Mn3—O13—C53—O12	12.3 (3)	C75—C70—C69—O17	173.2 (2)
Mn3—O13—C53—C54	−169.43 (13)	C69—C70—C71—C72	177.3 (2)
Mn4—O14—C61—O15	−1.2 (4)	C75—C70—C71—C72	−0.9 (3)
Mn4—O14—C61—C62	178.30 (14)	C69—C70—C75—C74	−176.7 (2)
Mn3—O15—C61—O14	−7.7 (3)	C71—C70—C75—C74	1.6 (3)
Mn3—O15—C61—C62	172.76 (13)	C70—C71—C72—C73	−1.2 (3)
Mn3—O17—C69—O16	8.6 (3)	C71—C72—C73—C74	2.7 (3)
Mn3—O17—C69—C70	−170.54 (13)	C71—C72—C73—C76	−175.6 (2)
Mn4—O19—C77—O18	10.9 (3)	C72—C73—C74—C75	−2.0 (4)
Mn4—O19—C77—C78	−169.13 (14)	C76—C73—C74—C75	176.3 (2)
Mn2—N1—C33—C34	−174.91 (15)	C70—C75—C74—C73	−0.1 (4)
C37—N1—C33—C34	0.7 (3)	O18—C77—C78—C79	−1.4 (3)
Mn2—N1—C37—C36	174.37 (15)	O18—C77—C78—C83	178.0 (2)
C33—N1—C37—C36	−1.5 (3)	O19—C77—C78—C79	178.6 (2)
Mn1—N2—C38—C39	166.40 (15)	O19—C77—C78—C83	−2.0 (3)
C42—N2—C38—C39	−1.7 (3)	C77—C78—C83—C82	−179.8 (2)
C44—N3—C43—O11^ii^	−170.87 (19)	C79—C78—C83—C82	−0.4 (4)
C44—N3—C43—C41	7.1 (3)	C80—C79—C78—C77	−179.9 (2)
C46—N3—C43—O11^ii^	1.0 (3)	C80—C79—C78—C83	0.7 (3)
C46—N3—C43—C41	178.99 (18)	C78—C79—C80—C81	−0.9 (4)
C43—N3—C44—C45	89.3 (3)	C82—C81—C80—C79	0.9 (4)
C46—N3—C44—C45	−82.6 (2)	C84—C81—C80—C79	−180.0 (2)
C43—N3—C46—C47	−72.9 (2)	C83—C82—C81—C80	−0.6 (4)
C44—N3—C46—C47	99.5 (2)	C83—C82—C81—C84	−179.8 (2)
C49—N4—C48—O10	0.3 (3)	C81—C82—C83—C78	0.4 (4)
C49—N4—C48—C36^ii^	178.03 (18)	C87—C86—C85—N5	1.1 (3)
C51—N4—C48—O10	−178.5 (2)	C95^i^—C86—C85—N5	177.86 (17)
C51—N4—C48—C36^ii^	−0.8 (3)	C88—C87—C86—C85	0.2 (3)
C48—N4—C49—C50	−77.2 (3)	C88—C87—C86—C95^i^	−176.26 (18)
C51—N4—C49—C50	101.6 (2)	N5—C89—C88—C87	0.9 (3)
C48—N4—C51—C52	97.0 (3)	C86—C87—C88—C89	−1.1 (3)
C49—N4—C51—C52	−81.8 (2)	C92—C91—C90—N6	−1.1 (3)
Mn3—N5—C85—C86	169.80 (15)	C90—C91—C92—C93	2.6 (3)
Mn3—N5—C89—C88	−170.47 (15)	C94—C93—C92—C91	−2.0 (3)
C85—N5—C89—C88	0.4 (3)	C100^ii^—C93—C92—C91	−178.93 (19)
C89—N5—C85—C86	−1.4 (3)	C92—C93—C94—N6	−0.3 (3)
Mn4—N6—C90—C91	174.16 (15)	C100^ii^—C93—C94—N6	176.89 (18)
Mn4—N6—C94—C93	−174.02 (15)	N7—C95—O20—Mn4	100.6 (3)
C94—N6—C90—C91	−1.1 (3)	C86^ii^—C95—O20—Mn4	−82.8 (3)
C90—N6—C94—C93	1.8 (3)	N8—C100—O21—Mn3	134.9 (2)
C96—N7—C95—O20	173.37 (19)	C93^i^—C100—O21—Mn3	−49.5 (3)

Symmetry codes: (i) *x*−1, *y*, *z*; (ii) *x*+1, *y*, *z*.

### Hydrogen-bond geometry (Å, °)

**Table:**

Cg1, Cg5, Cg10 and Cg12 are the centroids of the C2–C7, N1/C33–C37, C78–C83 and N6/C90–C94 rings, respectively.

**Table:**

*D*—H···*A*	*D*—H	H···*A*	*D*···*A*	*D*—H···*A*
O9—H9A···O7	0.94 (3)	1.62 (3)	2.552 (2)	170 (3)
O9—H9B···O5	0.94 (2)	1.59 (2)	2.520 (2)	173 (3)
O22—H22A···O16	0.92 (3)	1.64 (3)	2.544 (2)	166 (2)
O22—H22B···O18	0.97 (4)	1.60 (4)	2.558 (2)	169 (3)
C52—H52A···Cg12^ii^	0.96	2.87	3.782 (3)	160
C60—H60C···Cg1^iii^	0.96	2.99	3.841 (3)	149
C84—H84B···Cg10^iv^	0.96	2.88	3.609 (3)	134
C104—H10E···Cg5^i^	0.96	2.82	3.709 (3)	155

Symmetry codes: (ii) *x*+1, *y*, *z*; (iii) −*x*, −*y*, −*z*+1; (iv) −*x*+1, −*y*, −*z*+1; (i) *x*−1, *y*, *z*.
